# Diarylation of thiazolopyrimidines by laccase and their in vitro evaluation as antitumor agents

**DOI:** 10.1038/s41598-022-26820-9

**Published:** 2022-12-25

**Authors:** Mansour Shahedi, Rojina Shahani, Zohreh Habibi, Maryam Yousefi, Jesper Brask, Arash Minaei-Tehrani, Fatemeh Yazdi Samadi, Mehdi Mohammadi

**Affiliations:** 1 Department of Organic and Inorganic Chemistry, Faculty of Chemistry, Shahid Beheshti University, G.C., Tehran, Iran; 2 Nanobiotechnology Research Center, Avicenna Research Institute, ACECR, Tehran, Iran; 3 Novozymes A/S, Krogshøjvej 36, Bagsværd, 2880 Copenhagen, Denmark; 4 Bioprocess Engineering Department, Institute of Industrial and Environmental Biotechnology, National Institute of Genetic Engineering and Biotechnology (NIGEB), Tehran, Iran

**Keywords:** Green chemistry, Organic chemistry

## Abstract

A mild and efficient method was developed for the synthesis of new derivatives of thiazolo[3,2-a] pyrimidin-3(2H)-ones from available starting materials based on the oxidation of catechols to ortho-quinone by *Myceliophthora thermophila* laccase (Novozym 51,003) and 1,4-addition of active methylene carbon to these in situ generated intermediates in moderate to good yields (35–93%). The structure of the products was confirmed through ^1^H NMR, ^13^C NMR, HMBC, HSQC, DEPT-135, and mass spectroscopy techniques. These novel compounds were evaluated as active antitumor agents against human colorectal adenocarcinoma and liver adenocarcinoma cell lines. All compounds displayed potent inhibition activities against the HT-29 cell line with IC_50_ values of 9.8–35.9 µM, superior to the positive control doxorubicin, and most showed potent anticancer activities against the HepG2 cell line.

## Introduction

**Figure 1:**
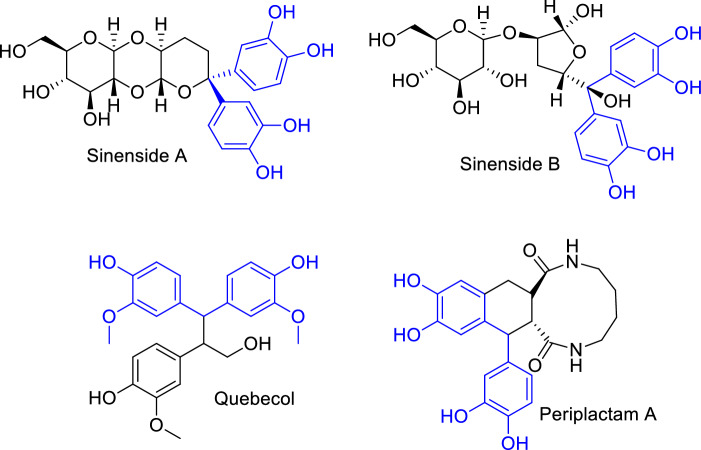
Natural products containing a carbon covalently bound to two catechols.

Carbon that is covalently attached to two catechols (1,2-dihydroxylated benzene ring) has always been of interest because of its potential biological activities and its presence in natural products^1–4^. Sinenside A and B (Fig. 1) that was isolated from the *Curculigo sinensis* plant showed antioxidant activity with IC_50_ values comparable to ascorbic acid^5^ (IC_50_ = 45.84 μM). Quebecol, formed during maple syrup production from *Acer saccharum’s* sap, had some anti-inflammatory properties^6^. Periplactam A (Fig. 1), isolated from *Periplaneta americana*, showed antiproliferation on human umbilical vein endothelial cells^7^ (HUVECs).

**Figure 2:**
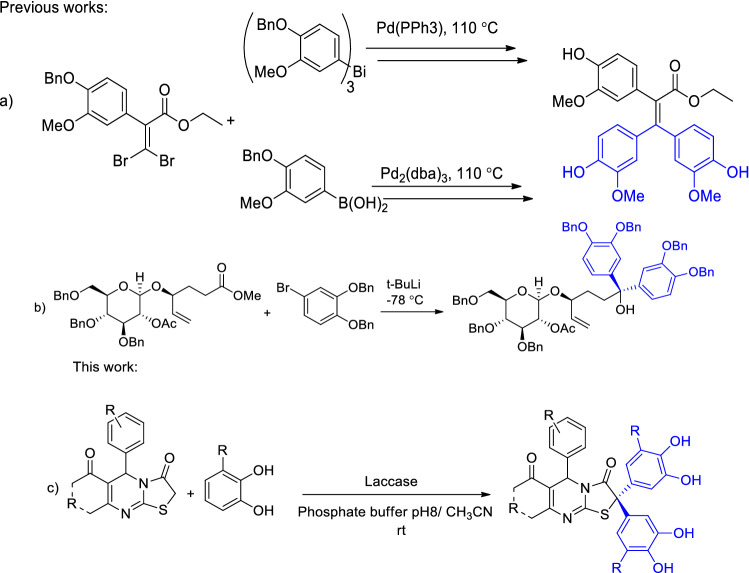
Previous works and this work.

These scaffolds have been conventionally prepared through boronic acid^8^, and bismuth^9^ substituted catechols used in C–C coupling reactions in the presence of palladium as a catalyst (Fig. 2a). The need for pre-functionalization of catechols, high temperature to perform the reaction, and obligation to protection and subsequent de-protection of hydroxyl groups are among the drawbacks of these methods. Alternatively, these structures can be prepared through halogen − metal exchange by employing *t-*BuLi and bromo catechol for addition to carbonyl groups (Fig. 2b)^10^. Disadvantages of this method include the requirement of low temperature (−78 °C) and the necessity of performing these reactions in the presence of nitrogen or argon atmosphere. An alternative method for preparing these scaffolds with mild and “green” reaction conditions and operational simplicity is therefore proposed (Fig. 2c).

Recently, laccases (benzenediol: O_2_ oxidoreductase E.C. 1.10.3.2.) which are multicopper oxidases, have attracted much attention in the synthesis of diverse organic compounds and degradation of environmentally related contaminants^11^. In this approach, oxygen acts as the oxidant, and as a result, water is formed. Laccases catalyze the oxidation of a wide range of phenolic and related compounds^12,13^. The application range of laccases can be further extended by using mediators. Laccases have been used in the aromatization of tetrahydroquinazolines^14^ and related *N*‐heterocyclic compounds^15^, oxidation of alcohols to aldehydes^16^ or carboxylic acids^17^ and trifluoromethylation of phenols^18^. Laccases are also utilized in domino processes such as catechol or hydroquinone oxidation to *ortho* or *para* quinone, respectively, and subsequent nucleophilic attack by -CH acids^19^ or -NH_2_^20^ or –SH^21^ nucleophiles. These one-pot reactions produce interesting organic compounds such as hydroxylated benzo[*b*]furans and pyrimidobenzothiazoles.

Thiazolopyrimidines and their derivatives possess various pharmacological properties such as anticancer^22^, antibacterial^23^, anti-inflammatory^24^. Thiazolo [3,2-a] pyrimidin-3(2H)-ones, due to the active methylene carbon at C-2, have been used as starting materials for synthesizing various organic compounds such as thioindigo-like compounds^25^, 2-benzylidene^26^, 2-arylhydrazono^27^ and 2-oxyimino^28^ derivatives. Up to now, application of laccases in preparation of new thiazolo [3,2-a] pyrimidin-3(2H)-one’s derivatives which include a gem-dicatechol has not been reported. Because of the simultaneous presence of catechol and thiazolopyrimidines, these hybrid compounds can have great potential in terms of biological properties.

## Result and discussion

Here we report a new strategy for the synthesis of gem-dicatechols through laccase-catalyzed oxidation of catechol to *ortho*-quinone and subsequent nucleophilic attack of active methylene carbon. First, 0.50 mmol of 6-acetyl-7-methyl-5-phenyl-2H-thiazolo[3,2-a] pyrimidin-3(5H)-one (**2a**) and 1 mmol of catechol (**3a**) as a model reaction were reacted in a mixture of phosphate buffer (0.01 M, pH 8) and CH_3_CN (2:1 v/v) at room temperature using a commercially available laccase (Novozym 51,003). The progress of the reaction was detected with TLC for 24 h. it turned out that the best reaction time was 4 h because the yield of product did not change after that. After workup with ethyl acetate and purification with silica gel column chromatography, product **4a** obtained in moderately good yield (65%) (Table 1 entry 2). As can be seen from Table 1, the model reaction was carried out in different buffer systems, such as citrate pH 3.5 and phosphate pH 4.5 and 7 with acetonitrile as a co-solvent. Reactions at pH 3.5 and 4.5 did not result in product and at pH 7, only 8% product was obtained. Also, different co-solvents, such as ethanol and methanol, were tested, but this did not result in product formation. Hence, the best condition was phosphate buffer pH 8 with acetonitrile. No product was observed when the reaction was tested without laccase (entry 4). The amount of enzyme was also optimized and the best yield was obtained when 1 ml of the commercial enzyme formulation was used for 0.1 mmol of substrate.

**Table1:** Optimization study.


Entry^a^	Enzyme (ml)	Buffer	Co-solvent	Time(h)	Yield^b^ (%)
1	0.5	Phosphate buffer pH 8 (10 mM)	CH_3_CN	4	23
2	1	Phosphate buffer pH 8 (10 mM)	CH_3_CN	4	65
3	2	Phosphate buffer pH 8 (10 mM)	CH_3_CN	4	63
4	0	Phosphate buffer pH 8 (10 mM)	CH_3_CN	24	N.R^c^
5	1	Phosphate buffer pH 8 (10 mM)	MeOH	24	N.R
6	1	Phosphate buffer pH 8 (10 mM	EtOH	24	N.R
7	1	Citrate buffer pH 3.5 (10 mM)	CH_3_CN	24	trace
8	1	Citrate buffer pH 3.5 (10 mM)	MeOH	24	N.R
9	1	Phosphate buffer pH 4.5 (10 mM)	MeOH	24	N.R
10	1	Phosphate buffer pH 7 (10 mM)	CH_3_CN	24	8
11	1	Phosphate buffer pH 4.5 (10 mM)	CH_3_CN	24	trace

^a^All reactions were performed using **2a** (0.5 mmol), **3a** (1 mmol) at ambient temperature. ^b^ Isolated yields. ^c^NR: no reaction.

**Figure 3:**
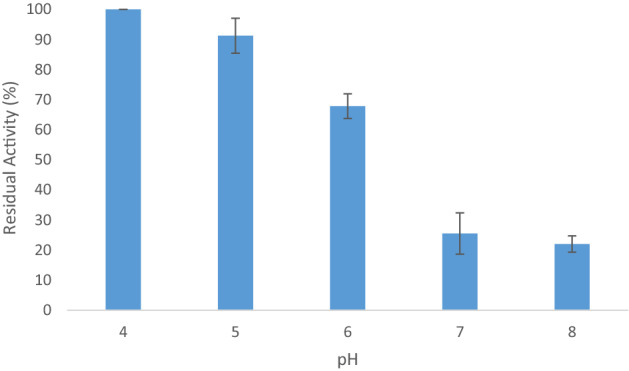
Effect of pH on laccase activity.

Laccase activity was measured at various pH (4.0–8.0). It was found that the best laccase activity was at pH 4.0–5.0 (Fig. 3). But the desired reaction could not be done at these pH values. This can be due to the lack of activation of the active methylene carbon at C-2 in acidic pH to attack the *ortho*-quinone.

**Figure 4:**
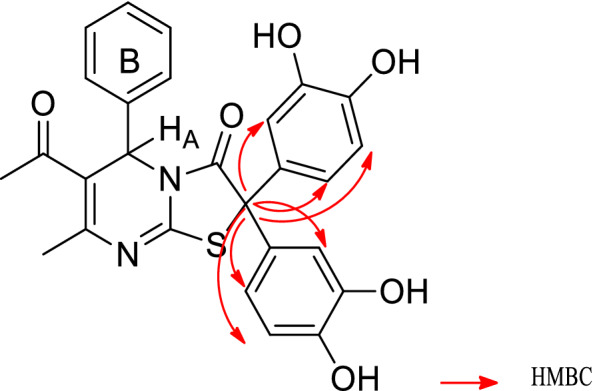
Correlation between quaternary carbon and protons in the catechol rings of **4a.**

For accurate characterization of the isolated products, various identification methods were used that included ^1^H NMR, ^13^C NMR, H, H-COSY, HMBC, HSQC, DEPT-135 and mass spectroscopy. The DEPT-135 and HSQC spectrum (supporting information) of **4a** exhibited the existence of a quaternary carbon (δ_c_ 66.94 ppm) representing the carbon attached to two catechol molecules. Based on the HMBC spectrum of **4a** (supporting information) the correlation between the quaternary carbon (δ_c_ 66.94 ppm) and the catechol protons is well illustrated (Fig. 4).

Also, H, H-COSY spectrum of **4a** revealed the presence of three spin systems as follows: one between protons of a catechol ring at δ_H_ 6.09–6.12 (m, 1H) that overlapped with H_A_ (Fig. 4) and two doublets at δ_H_ 6.44 (d, *J* = 2.4 Hz, 1H) and δ_H_ 6.47 (d, *J* = 8.4 Hz, 1H), while the second spin system occurred between protons of another catechol ring at δ_H_ 6.69 (dd, *J* = 8.3, 2.5 Hz, 1H) and δ_H_ 6.75 (m, 2H). The third spin system δ_H_ 7.32 (m, 5H) is related to the phenyl ring B.

After optimization and characterization, the scope of the reaction was explored and the results are summarized in Table 2. The results demonstrated a relatively broad tolerance for substitutions on the thiazolopyrimidine system. Results showed that compounds with ketone substituents on the 1,2-dihydropyrimidine ring had better yields than ester substituents on this ring **4 h** (73%) and **4c** (82%) versus **4d** (35%) and **4e** (79%), respectively. Only thiazolo [3,2-a] pyrimidin-3(2H)-ones that in position 5 contain phenyl-4-Cl (**4 l**, trace), phenyl-4-Me (**4 m**, trace) and vanilline (**4d,** 35%) resulted in low yields.

**Table 2:** Scope of reaction catalyzed by laccase. All reactions were performed using **2** (0.5 mmol), and **3** (1 mmol) at room temperature.

**Figure 5:**
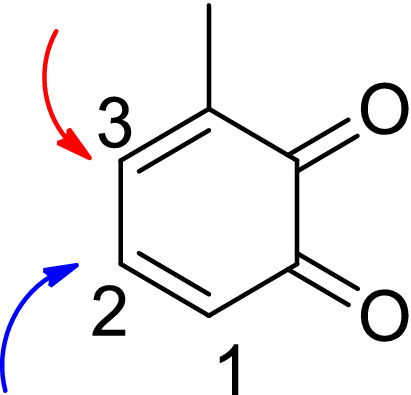
Regioselectivity of the laccase-catalyzed reaction between 3-methyl catechol **3b** and **2e**, **2 k.**

To explore further reaction diversity on the catechol side, 3-methyl catechol **3b** was used. As shown in Table 2, this resulted in product formation with excellent yields, even better than with catechol **3a** (compare **4o** (93%) vs **4e** (79%) and **4n** (83%) vs. **4 k** (68%)). When 3-methyl catechol **3b** was used, it was expected that a mixture of products resulting from the nucleophilic attack on two sites (carbon 2 and 3 in Fig. 5) would be observed. However, according to the coupling constant (*J* = 2.4 Hz), which is caused by meta-coupling, just one product was observed that resulted from a nucleophilic attack on carbon 2.

**Figure 6:**
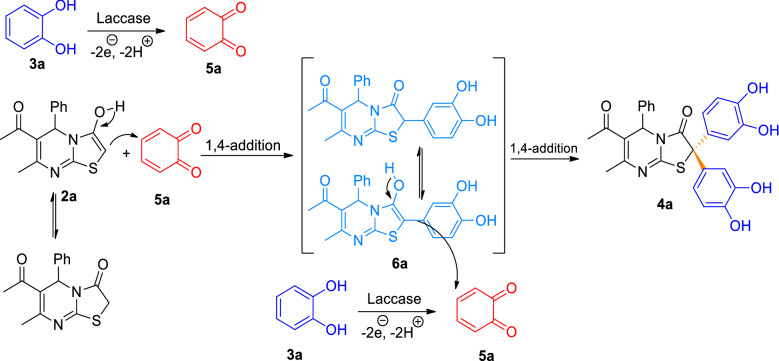
Proposed mechanism.

Based on previous studies and control experiments, a possible mechanism can be proposed. It is assumed that the reaction starts with the laccase-catalyzed oxidation of catechol (**3a**) to *o*-benzoquinone (**5a**) (Fig. 6). This is followed by a 1,4-addition of the active methylene carbon and the formation of the intermediate (**6a**). Oxidation of the second catechol to *o*-benzoquinone and nucleophilic attack of the intermediate **6a** produces the final product **4a.**

Some features of this method are consistent with the principles of green chemistry^29^: this reaction was carried out in a phosphate buffer as a solvent and acetonitrile as a co-solvent with a ratio of 2:1, which is considered a safe environment. Also, in terms of energy efficiency, this reaction is done at room temperature. In this method, there is no need to protect functional groups and therefore reduce derivatives.

To evaluate the antitumor potency of prepared compounds (**4a-d,4f.-k** and **4o**), their in vitro cytotoxic activity was evaluated against two different cancer cell lines HT-29 (human colorectal adenocarcinoma) and HepG2 (liver adenocarcinoma) using the 3-(4,5-dimethylthiazol-2-yl)-2,5-diphenyltetrazolium bromide (MTT) assay^30^. HT-29 and HepG2 were treated with varying concentrations of the prepared compounds for 48 h. Doxorubicin was used as a positive control (IC_50_ = 63 ± 2.97 μM for HT-29 and 69 ± 3.71 μM for HepG2 at 48 h). The antiproliferative efficacy data are presented as IC_50_ values defined as the concentration of the compound that decreases cell proliferation at 50%. The IC_50_ values were generated from at least three independent experiments.

IC_50_ values for the cytotoxic effects of the various synthetic compounds (**4a-d,4f.-k,** and **4o**) are shown in Table 3. All compounds **4a-d,4f.-k,** and **4o** displayed potent inhibition activities against HT-29 cell lines. Among the compounds tested, compounds **4b**, **4d**, **4 k**, and **4o** stood out as more potent (IC_50_ = 17.1 ± 2.66 µM for **4b**, IC_50_ = 11.5 ± 1.38 µM for **4d**, IC_50_ = 11.1 ± 0.83 µM for **4 k**, IC_50_ = 9.8 ± 1.41 µM for **4o**), significantly better than the positive control doxorubicin. Generally, structure–activity relationships (SAR) at the cellular level for these thiazolopyrimidine derivatives demonstrated that compounds with ester substituents on 1,2-dihydropyrimidine ring seem more potent than those with ketone substituents, comparing compounds **4d, 4 k** to compounds **4 h, 4j** respectively.

**Table 3:** In vitro anticancer screening of the compounds against HT(29) and HepG2 cancer cells expressed as IC_50_ values (μM).

Compounds	IC_50_(µM)	
	HT-29	HepG2
4a	35.4 ± 4.24	67 ± 3.62
4b	17.1 ± 2.66	22 ± 2.38
4c	42.7 ± 4.34	˃120
4d	11.5 ± 1.38	31.2 ± 2.63
4f.	21.2 ± 2.32	22.9 ± 2.75
4 g	21.2 ± 2.18	10.6 ± 0.67
4 h	35.9 ± 3.96	48.2 ± 2.96
4i	23.8 ± 3.10	49.2 ± 2.93
4j	21.9 ± 2.67	45.9 ± 4.08
4 k	11.1 ± 0.83	˃120
4o	9.8 ± 1.41	11.7 ± 2.11
Doxorubicin	63 ± 2.97	69 ± 3.71

Besides, it was worth mentioning that all target compounds except **4c** and **4 k** showed potent anticancer activities against the HepG2 cell line and were more potent than the positive control doxorubicin. It is noteworthy that **4o** showed excellent results with both HT-29 and HepG2.

## Conclusions

To summarize, a simple and efficient method was developed to synthesize new derivatives of thiazolopyrimidin-3(2H)-ones through oxidation of catechols by laccase to related *o*-quinones and nucleophilic attack of active methylene carbon of thiazolopyrimidin-3(2H)-ones to these intermediates. The reaction was performed at room temperature and used aerial oxygen as an oxidant. These novel compounds were evaluated as active antitumor agents against human colorectal adenocarcinoma and liver adenocarcinoma cell lines. All compounds displayed potent inhibition activities against the HT-29 cell line with IC_50_ values of 9.8–35.9 µM, superior to the positive control doxurobicin, and most showed potent anticancer activities against the HepG2 cell line. Future mechanistic studies are unambiguously needed to elucidate anticancer effects of our compounds, providing an improved basis for its prediction in further clinical studies.

## Exprimental

### General remarks

All reagents are commercially available and used without further purification. Solvents used for extraction and purification were distilled befor use. Laccase (Novozym 51,003) was a generous gift from Novozymes (Copenhagen, Denmark). Reactions were monitored by thin-layer chromatography (TLC) using silica gel 60 F_254_. ^1^H and ^13^C NMR spectra were recorded at 300 (75), 400 (100), and 500 (125) MHz on Bruker Avance spectrometers using DMSO-*d6* and acetone-*d6* as solvents. The chemical shifts were referenced to the solvent signals at δH/C 2.49/ 39.50 ppm (DMSO-*d*_*6*_) and δH/C δH/C 2.05/29.85 ppm (acetone-*d*_*6*_) relative to TMS as internal standards. Melting points were determined with a melting point Thermo Scientific 9100 apparatus and are uncorrected. Mass spectra were recorded with an Agilent Technologies (HP) 5975C mass spectrometer by electron ionization (EI) (20–70 eV). Elemental analyses were performed on an elementar analysensysteme GmbH VarioEL CHN mode.

### Determination of the optimum pH activity:

100 µl of laccase solution was added to 2 ml of buffers at different pH levels (4.0–8.0) and incubated at 25 °C and 200 rpm for 24 h. the activities of samples were measured at the same pH levels.

### The activity of laccase

The activity was measured spectrophotometrically (Thermo Electron, model UV1 spectrophotometer) with ABTS as substrate (1 mM) in 100 mM sodium citrate buffer pH 4.5. To measure the laccase activity, 5 μl of enzyme solution was added to 965 μl of related buffer and 30 μl of the ABTS (1 mM) solution at room temperature to start the oxidation reaction. The change in absorbance at 420 nm (ε = 36 × 10^3^ M^−1^ cm^−1^) was recorded for 40 s (per 5 s) and the catalytic activity was assayed by calculating the slope of the initial linear segment of the kinetic curve^31^. One unit of enzyme activity was defined as the amount of enzyme required to oxidize 1 μmol of ABTS per min and the activities were expressed in U/g.

#### Synthesis of dihydropyrimidines (DHPMs)

According to the literature procedure^32^ , DHPMs **1a-1**i and **1j-1 m** were prepared by the following method. A mixture of benzaldehyde derivatives (5 mmol), acetylacetone or ethyl acetoacetate (5 mmol), thiourea (7.5 mmol), and NH_4_Cl (0.8 mmol) was heated with stirring at 100 °C for 3 h. After cooling, the reaction mixture was washed with cold water and the residue recrystallized from ethanol.

DHPMs **1f.-1 h** were prepared by a mixture of benzaldehyde derivatives (5 mmol), dimedone (5 mmol), thiourea (7.5 mmol) and a few drop H_2_SO_4_ in ethanol (15 ml) was heated under reflux overnight. After cooling, the reaction mixture was dropped in cold water and the precipitated residue recrystallized from ethanol.

#### Synthesis of thiazolopyrimidin-3(2H)-ones (2a-m)

According to the literature procedure^28^ a mixture of corresponding DHPMs (1 mmol) and ethyl chloroacetate (1 ml, 90 mmol) was heated for 30 min at 110–115° C. The solution was cooled, and the precipitate was filtered off and washed with EtOAc. The prepared hydrochloride was dissolved in EtOH (10 mL) and ammonia was added to adjust pH 7.5–8.0. Evaporation of the solvent at the reduced pressure, followed evaporation from the chloroform solution gave pure compounds **2a-m**.

#### 6-acetyl-7-methyl-5-(3-nitrophenyl)-2H-thiazolo[3,2-a]pyrimidin-3(5H)-one (2c)

Solid (brown), Melting point: 179–181 °C, Isolated yield = 92%, ^1^H NMR (300 MHz, DMSO-*d*_6_) δ 8.12 (m, 2H), 7.79–7.56 (m, 2H), 6.13 (s, 1H), 4.16 (m, 2H), 2.43 (s, 3H), 2.27 (s, 3H). ^13^C NMR (75 MHz, DMSO-*d*_6_) δ 196.3, 171.5, 149.0, 148.1, 141.8, 135.0, 130.8, 124.0, 123.1, 116.3, 54.5, 33.9, 31.6, 22.8.

#### 8,8-dimethyl-5-phenyl-8,9-dihydro-2H-thiazolo[2,3-b]quinazoline-3,6(5H,7H)-dione (2f.)

Solid (yellow), Melting point: 125–127 °C, Isolated yield = 89%, ^1^H NMR (300 MHz, DMSO-*d*_6_) δ 7.41–7.17 (m, 5H), 5.85 (s, 1H), 4.18 (s, 2H), 2.52 (d, *J* = 8.0 Hz, 2H), 2.23 (d, *J* = 16.1 Hz, 1H), 2.11 (d, *J* = 16.2 Hz, 1H), 1.02 (s, 3H), 0.91 (s, 3H). ^13^C NMR (75 MHz, DMSO-*d*_*6*_) δ 195.3, 171.6, 165.6, 155.5, 140.6, 128.9, 128.7, 127.8, 113.5, 53.5, 50.5, 43.8, 33.3, 32.8, 28.8, 27.5.

### Typical experimental procedure for the synthesis of product 4

A 100 mL round bottom flask with a magnetic stir bar was charged with a solution or suspension of a catechol 3 (1 mmol) and a 2 (0.50 mmol) in acetonitrille (8 mL). Phosphate buffer (0.10 mM, pH 8.0, 16 mL) and *Myceliophthora thermophila* laccase (Novozyme 51,003) (5 ml, 2.47 g, 5000 U) were added and the mixture was stirred under air. The reaction progress was detected with thin-layer chromatography until thiazolo[3,2-a] pyrimidine was completely consumed (4 h). Then the reaction mixture was diluted with EtOAc. The layers were decanted and the aqueous phase was extracted with EtOAc (3 × 20 mL). The combined organic layers were washed with brine, dried over anhydrous Na_2_SO_4_ and filtered, and removed the solvents in vacuo. Purification by preparative thin layer chromatography (Hexane: EtOAc) (3:1) provided target compound **4**.

### MTT assay

The prepared compounds (**4a-d,4f.-k** and **4o**) were examined to assess their cytotoxicity effects against two cancer cell lines (human colorectal adenocarcinoma cell line HT-29 and liver adenocarcinoma cell line HepG2) using a standard MTT-based colorimetric assay. These cells were cultured in RPMI 1640 medium supplemented with 10% heat-activated fetal bovine serum (FBS). A day before treatment, appropriate numbers of cells were seeded into each well of 96-well plates (Corning, New York, USA) and incubated at 37 °C with 5% CO_2_. Testing compounds at pre-set concentrations were added to each well with doxorubicin being used as a positive control. Each concentration was in triplicate. After 48 h exposure period, MTT reagent was added (0.5 mg/mL) to the wells and incubated for the next 1 to 2 h at 37 °C, then the medium was replaced by DMSO to dissolve the purple formazan crystals formed. The optical absorbance of each well was determined by an ELISA plate reader at 570 nm.

### Characterization of products 4a-k, 4n ,4o

#### 6-acetyl-2,2-bis(3,4-dihydroxyphenyl)-7-methyl-5-phenyl-2H-thiazolo[3,2-a]pyrimidin-3(5H)-one (4a)

Solid (light brown), Melting point: 125–127 °C (decompose), 165 mg, Isolated yield = 65%, ^1^H NMR (300 MHz, DMSO-*d*_6_) δ 9.28–8.96 (m, 4H), 7.30 (q, *J* = 7.3 Hz, 5H), 6.83–6.60 (m, 3H), 6.55–6.38 (m, 2H), 6.09 (d, *J* = 8.5 Hz, 2H), 2.36 (s, 3H), 2.26 (s, 3H). ^13^C NMR (75 MHz, DMSO-*d*_*6*_) δ 197.0, 173.1, 157.0, 150.1, 146.1, 145.8, 145.6, 145.5, 140.1, 130.9, 130.1, 129.1, 129.0, 127.8, 119.6, 118.9, 117.9, 116.2, 115.7, 115.3, 67.4, 55.3, 31.1, 23.8. MS (EI, 70 eV): m/z = 502 [M^+^]. Anal. Calcd for C_27_H_22_N_2_O_6_S: C 64.53; H 4.41; N 5.57; Found: C 64.22; H 4.21; N 4.48.

#### 6-acetyl-2,2-bis(3,4-dihydroxyphenyl)-7-methyl-5-(p-tolyl)-2H-thiazolo[3,2-a]pyrimidin-3(5H)-one (4b)

Solid (brown), Melting point: 230–232 °C, 145 mg, Isolated yield = 56%, ^1^H NMR (300 MHz, DMSO- *d*_*6*_) δ 9.17 (s, 4H), 7.14 (s, 4H), 6.70 (m, 3H), 6.55–6.38 (m, 2H), 6.12 (m, 2H), 2.34 (s, 3H), 2.25 (m, 6H).^13^C NMR (75 MHz, DMSO-*d*_*6*_) δ 197.1, 173.1, 156.9, 149.9, 146.1, 145.8, 145.6, 145.5, 138.4, 137.2, 130.9, 130.2, 129.7, 127.7, 119.5, 119.0, 117.9, 116.2, 116.1, 115.7, 115.3, 67.4, 55.0, 31.0, 23.7, 21.2. MS (EI, 70 eV): m/z = 516 [M^+^]. Anal. Calcd for C_28_H_24_N_2_O_6_S: C 65.10; H 4.68; N 5.42; Found: C 65.43; H 4.34; N 5.12.

#### 6-acetyl-2,2-bis(3,4-dihydroxyphenyl)-7-methyl-5-(3-nitrophenyl)-2H-thiazolo[3,2-a]pyrimidin-3(5H)-one (4c)

Solid (light brown), Melting point: 85–87 °C(decompose), 225 mg, Isolated yield = 82%, ^1^H NMR (300 MHz, DMSO-*d*_6_) δ 9.44–8.86 (m, 4H), 8.16 (d, *J* = 8.0 Hz, 1H), 8.05 (s, 1H), 7.67 (m, 2H), 6.82–6.59 (m, 3H), 6.47 (d, *J* = 8.3 Hz, 1H), 6.36 (d, *J* = 2.6 Hz, 1H), 6.25–6.12 (m, 2H), 2.42 (s, 3H), 2.31 (s, 3H). ^13^C NMR (75 MHz, DMSO-*d*_6_) δ 196.7, 173.0, 157.5, 151.6, 148.0, 146.1, 145.9, 145.6, 145.5, 142.1, 134.3, 130.9, 130.4, 129.58, 123.9, 122.3, 119.6, 118.9, 117.3, 116.2, 116.0, 115.8, 115.3, 67.5, 54.7, 31.6, 24.2. MS (EI, 70 eV): m/z = 439 [M^+^-catechol]. Anal. Calcd for C_27_H_21_N_3_O_8_S: C 59.23; H 3.87; N 7.67; Found: C 59.12; H 3.71; N 7.51.

#### Ethyl 2,2-bis(3,4-dihydroxyphenyl)-5-(4-hydroxy-3-methoxyphenyl)-7-methyl-3-oxo-3,5-dihydro-2H-thiazolo[3,2-a]pyrimidine-6-carboxylate (4d)

Solid (light brown), Melting point: 176–178 °C, 102 mg, Isolated yield = 35%, ^1^H NMR (300 MHz, DMSO-*d*_6_) δ 9.17 (s, 5H), 6.69 (m, 6H), 6.52 (m, 2H), 6.23 (d, *J* = 8.3 Hz, 1H), 5.89 (s, 1H), 4.07 (q, *J* = 7.2 Hz, 2H), 3.66 (s, 3H), 2.33 (s, 3H), 1.16 (t, *J* = 7.2 Hz, 3H).^13^C NMR (75 MHz, DMSO-*d*_6_) δ 173.2, 165.4, 157.3, 151.2, 147.7, 147.2, 146.0, 145.9, 145.6, 145.5, 131.8, 130.8, 130.3, 119.8, 119.5, 119.1, 116.3, 116.1, 115.9, 115.7, 115.3, 111.6, 109.1, 67.4, 60.5, 55.9, 55.1, 22.7, 14.4. MS (EI, 70 eV): m/z = 551 [M^+^-Et], 470 [M^+^-catechol]. Anal. Calcd for C_29_H_26_N_2_O_9_S: C 60.20; H 4.53; N 4.84; Found: C 60.32; H 4.18; N 4.79.

#### Ethyl 2,2-bis(3,4-dihydroxyphenyl)-7-methyl-5-(3-nitrophenyl)-3-oxo-3,5-dihydro-2H-thiazolo[3,2-a]pyrimidine-6-carboxylate (4e)

Solid (light brown), Melting point: 103–105 °C(decompose), 228 mg, Isolated yield = 79%, ^1^H NMR (300 MHz, DMSO-*d*_6_) δ 9.20 (s, 4H), 8.18 (d, *J* = 7.9 Hz, 1H), 8.05 (s, 1H), 7.70 (m, 2H), 6.71 (m, 3H), 6.49 (d, *J* = 8.3 Hz, 1H), 6.39 (s, 1H), 6.24 (d, *J* = 8.4 Hz, 1H), 6.12 (s, 1H), 4.04 (q, *J* = 7.2 Hz, 2H), 2.39 (s, 3H), 1.13 (t, *J* = 7.2 Hz, 3H). ^13^C NMR (75 MHz, DMSO-*d*_6_) δ 172.9, 164.9, 152.9, 147.8, 146.1, 145.9, 145.6, 145.5, 142.5, 134.2, 130.9, 130.2, 129.7, 123.9, 122.4, 119.6, 119.0, 116.2, 115.7, 115.3, 107.8, 67.4, 60.7, 55.1, 23.0, 14.2. MS (EI, 70 eV): m/z = 549 [M^+^-Et]. Anal. Calcd for C_28_H_23_N_3_O_9_S: C 58.23; H 4.01; N 7.28; Found: C 58.62; H 4.21; N 7.48.

#### 2,2-bis(3,4-dihydroxyphenyl)-8,8-dimethyl-5-phenyl-8,9-dihydro-2H-thiazolo[2,3-b]quinazoline-3,6(5H,7H)-dione (4f.)

Solid (light brown), Melting point: 144–146 °C(decompose), 196 mg, Isolated yield = 61%, ^1^H NMR (499 MHz, Acetone-d6) δ 8.04 (m, 2H), 7.34 (d, *J* = 7.9 Hz, 2H), 7.29 (t, *J* = 7.4 Hz, 2H), 7.27–7.23 (m, 1H), 6.90–6.86 (m, 1H), 6.83 (s, 2H), 6.63–6.55 (m, 2H), 6.34 (dd, *J* = 8.3, 2.4 Hz, 1H), 6.04 (s, 1H), 2.62–2.49 (m, 2H), 2.25 (d, *J* = 16.2 Hz, 1H), 2.15 (d, *J* = 16.2 Hz, 1H), 1.08 (s, 3H), 0.98 (s, 3H).^1^H NMR (300 MHz, DMSO-*d*_6_) δ 9.12 (s, 4H), 7.25 (m, 5H), 6.81–6.53 (m, 3H), 6.47 (d, *J* = 8.6 Hz, 2H), 6.15 (d, *J* = 6.9 Hz, 1H), 5.91 (s, 1H), 2.29–2.04 (m, 2H), 1.00 (s, 3H), 0.88 (s, 3H). ^13^C NMR (75 MHz, DMSO-*d*_6_) δ 195.5, 173.1, 160.6, 156.4, 146.1, 145.9, 145.5, 140.4, 130.6, 129.9, 128.8, 128.6, 127.5, 119.6, 118.9, 116.2, 115.8, 115.3, 114.5, 67.3, 53.9, 50.6, 44.5, 32.7, 29.0, 27.3. MS (EI, 70 eV): m/z = 542 [M^+^]. Anal. Calcd for C_30_H_26_N_2_O_6_S: C66.41; H 4.83; N 5.16; Found: C 66.22; H 4.51; N 5.48.

#### 5-(4-chlorophenyl)-2,2-bis(3,4-dihydroxyphenyl)-8,8-dimethyl-8,9-dihydro-2H-thiazolo[2,3-b]quinazoline-3,6(5H,7H)-dione (4 g)

Solid (brown), Melting point: 164–166 °C(decompose), 151 mg, Isolated yield = 52%, ^1^H NMR (499 MHz, Acetone-*d*_6_) δ 8.20 (s, 1H), 8.13 (m, 2H), 8.00 (s, 1H), 7.35 (m, 4H), 6.90–6.79 (m, 3H), 6.65 (dd, *J* = 8.3, 1.5 Hz, 1H), 6.60 (t, *J* = 1.9 Hz, 1H), 6.40 (dt, *J* = 8.4, 1.9 Hz, 1H), 6.03 (s, 1H), 2.63–2.52 (m, 2H), 2.29–2.17 (m, 2H), 1.09 (s, 3H), 0.99 (s, 3H). ^1^H NMR (300 MHz, DMSO-d_*6*_) δ 9.20 (s, 4H), 7.37 (d, *J* = 8.0 Hz, 2H), 7.27 (d, *J* = 8.2 Hz, 2H), 6.81–6.60 (m, 3H), 6.59–6.41 (m, 2H), 6.20 (d, *J* = 8.4 Hz, 1H), 5.92 (s, 1H), 2.33–2.06 (m, 2H), 1.02 (s, 3H), 0.89 (s, 3H).^13^C NMR (75 MHz, DMSO-d_*6*_) δ 195.5, 173., 160.61, 156.5, 146.1, 146.0, 145.6, 139.3, 133.2, 130.5, 129.7, 129.5, 128.9, 119.7, 118.9, 116.2, 115.8, 115.3, 114.1, 67.2, 53.5, 50.5, 44.5, 32.7, 28.9, 27.3. MS (EI, 70 eV): m/z = 576 [M^+^]. Anal. Calcd for C_30_H_25_ClN_2_O_6_S: C 62.44; H 4.37; N 4.85; Found: C 62.11; H 4.30; N 4.61.

#### 2,2-bis(3,4-dihydroxyphenyl)-5-(4-hydroxy-3-methoxyphenyl)-8,8-dimethyl-8,9-dihydro-2H-thiazolo[2,3-b]quinazoline-3,6(5H,7H)-dione (4 h)

Solid (light yellow), 215 mg, Isolated yield = 73%, Melting point: 176–178 °C, ^1^H NMR (499 MHz, Acetone-*d*_6_) δ 8.15 (s, 3H), 8.01 (s, 1H), 7.66 (s, 1H), 6.93–6.88 (m, 2H), 6.84 (d, *J* = 1.4 Hz, 2H), 6.80 (dd, *J* = 8.1, 2.0 Hz, 1H), 6.75 (d, *J* = 8.2 Hz, 1H), 6.67–6.61 (m, 2H), 6.39 (dd, *J* = 8.4, 2.4 Hz, 1H), 6.00 (s, 1H), 3.76 (s, 3H), 2.59 (m, 1H), 2.57–2.49 (m, 1H), 2.27 (d, *J* = 16.2 Hz, 1H), 2.19 (d, *J* = 16.2 Hz, 1H), 1.09 (s, 3H), 1.02 (s, 3H). ^1^H NMR (300 MHz, DMSO-*d*_*6*_) δ 9.17 (s, 5H), 6.71 (m, 6H), 6.53 (m, 2H), 6.22 (d, *J* = 8.2 Hz, 1H), 5.87 (s, 1H), 3.67 (s, 3H), 2.46 (s, 2H), 2.31–2.05 (m, 2H), 1.04 (s, 3H), 0.94 (s, 3H).^13^C NMR (75 MHz, DMSO-*d*_*6*_) δ 195.6, 173.3, 160.4, 156.2, 147.6, 147.0, 146.1, 145.9, 145.6, 131.5, 130.8, 130.0, 120.0, 119.6, 118.9, 116.2, 116.1, 115.7, 115.3, 114.6, 111.7, 67.3, 56.0, 53.4, 50.6, 44.4, 32.7, 29.1, 27.2. MS (EI, 70 eV): m/z = 480 [M^+^-catechol]. Anal. Calcd for C_31_H_28_N_2_O_8_S: C 63.25; H 4.79; N 4.76; Found: C 63.65; H 4.51; N 4.38.

#### 6-acetyl-2,2-bis(3,4-dihydroxyphenyl)-7-methyl-5-(4-nitrophenyl)-2H-thiazolo[3,2-a]pyrimidin-3(5H)-one (4i)

Solid (light brown), Melting point: 140–142 °C, 247 mg, Isolated yield = 90%, ^1^H NMR (300 MHz, DMSO-*d*_*6*_) δ 9.35–8.95 (m, 4H), 8.19 (d, *J* = 8.3 Hz, 2H), 7.54 (d, *J* = 8.3 Hz, 2H), 6.71 (m, 3H), 6.50 (m, 1H), 6.37 (s, 1H), 6.18 (m, 2H), 2.40 (s, 3H), 2.30 (s, 3H). ^13^C NMR (75 MHz, DMSO-*d*_*6*_) δ 196.6, 172.9, 157.4, 151.3, 147.6, 146.9, 146.1, 145.9, 145.6, 145.5, 130.4, 129.6, 129.2, 124.3, 119.6, 119.0, 117.4, 116.1, 116.0, 115.7, 115.4, 67.5, 54.7, 31.5, 24.1. (EI, 70 eV): m/z = 439 [M^+^- catechol]. Anal. Calcd for C_28_H_23_N_3_O_9_S: C 58.23; H 4.01; N 7.28; Found: C 58.43; H 4.11; N 7.03.

#### 6-acetyl-2,2-bis(3,4-dihydroxyphenyl)-5-(4-methoxyphenyl)-7-methyl-2H-thiazolo[3,2-a]pyrimidin-3(5H)-one (4j)

Solid (light yellow), Melting point: 134–136 °C(decompose), 227 mg, Isolated yield = 85%, ^1^H NMR (300 MHz, DMSO-*d*_6_) δ 9.26–9.13 (m, 3H), 9.04 (s, 1H), 7.25–7.15 (m, 2H), 6.89 (m, 2H), 6.84–6.62 (m, 3H), 6.52–6.37 (m, 2H), 6.13–6.03 (m, 2H), 3.42 (s, 3H), 2.35 (s, 3H), 2.23 (s, 2H). ^13^C NMR (75 MHz, DMSO-*d*_6_) δ 164.4, 154.6, 150.7, 150.5, 150.2, 136.8, 135.7, 134.0, 124.2, 123.6, 120.8, 120.4, 120.0, 119.1, 72.0, 60.2, 59.5, 45.4, 45.1, 44.8, 44.5, 44.3, 44.0, 43.7, 35.7, 28.4. MS (EI, 70 eV): m/z = 424 [M^+^-catechol]. Anal. Calcd for C_28_H_24_N_2_O_7_S: C 63.15; H 4.54; N 5.26; Found: C 63.05; H 4.24; N 5.16.

#### Ethyl 2,2-bis(3,4-dihydroxyphenyl)-5-(4-methoxyphenyl)-7-methyl-3-oxo-3,5-dihydro-2H-thiazolo[3,2-a]pyrimidine-6-carboxylate (4 k)

Solid (light brown), Melting point: 100–102 °C(decompose), 192 mg, Isolated yield = 68%, ^1^H NMR (300 MHz, DMSO-*d*_*6*_) δ 9.52–8.95 (m, 4H), 7.17 (d, J = 7.9 Hz, 2H), 6.88 (d, *J* = 7.2 Hz, 3H), 6.72 (s, 2H), 6.46 (m, 2H), 6.15 (d, *J* = 8.1 Hz, 1H), 5.91 (s, 1H), 4.03 (q, *J* = 7.2 Hz, 2H), 3.72 (s, 3H), 2.35 (s, 3H), 1.13 (t, *J* = 7.1 Hz, 3H).^13^C NMR (75 MHz, DMSO-_d*6*_) δ 164.3, 150.5, 150.3, 150.1, 133.6, 123.7, 120.4, 119.0, 113.6, 65.2, 60.2, 45.3, 45.0, 44.8, 44.5, 44.2, 43.9, 43.7, 27.5, 19.0. MS (EI, 70 eV): m/z = 454 [M^+^- catechol]. Anal. Calcd for C_29_H_26_N_2_O_8_S: C 61.91; H 4.66; N 4.98; Found: C 61.71; H 4.16; N 4.68.

#### Ethyl 2,2-bis(3,4-dihydroxy-5-methylphenyl)-5-(4-methoxyphenyl)-7-methyl-3-oxo-3,5-dihydro-2H-thiazolo[3,2-a]pyrimidine-6-carboxylate (4n)

Solid (light yellow), Melting point: 146–148 °C(decompose), 245 mg, Isolated yield = 83%, ^1^H NMR (300 MHz, DMSO-*d*_6_) δ 9.19 (s, 4H), 7.19 (d, *J* = 8.2 Hz, 2H), 6.90 (d, *J* = 8.3 Hz, 2H), 6.64 (s, 2H), 6.33–6.26 (m, 1H), 5.90 (s, 1H), 5.81 (s, 1H), 4.02 (q, *J* = 7.2 Hz, 2H), 3.72 (s, 3H), 2.50 (s, 3H), 2.09 (s, 3H), 1.81 (s, 3H), 1.12 (t, *J* = 7.1 Hz, 3H). ^13^C NMR (75 MHz, DMSO-*d*_6_) δ 177.8, 170.0, 164.4, 162.2, 156.5, 149.6, 148.7, 148.4, 137.4, 135.5, 133.8, 133.3, 129.4, 129.0, 125.9, 124.9, 118.9, 118.6, 118.4, 113.4, 72.1, 65.2, 60.2, 59.6, 45.4, 45.1, 44.8, 44.6, 44.3, 44.0, 43.7, 27.5, 21.3, 21.0, 19.0. (EI, 70 eV): m/z = 590 [M^+^]. Anal. Calcd for C_31_H_30_N_2_O_8_S: C 63.04; H 5.12; N 4.74; Found: C 62.74; H 5.02; N 4.24.

#### Ethyl 2,2-bis(3,4-dihydroxy-5-methylphenyl)-7-methyl-5-(3-nitrophenyl)-3-oxo-3,5-dihydro-2H-thiazolo[3,2-a]pyrimidine-6-carboxylate (4o)

Solid (light yellow), Melting point: 96–98 °C(decompose), 282 mg, Isolated yield = 93%, ^1^H NMR (300 MHz, DMSO-*d*_*6*_) δ 9.36 (d, 2H), 8.51 (d, 2H), 8.19 (d, *J* = 8.5 Hz, 1H), 8.06 (s, 1H), 7.71 (m, 2H), 6.65 (d, *J* = 2.5 Hz, 1H), 6.59 (d, *J* = 2.4 Hz, 1H), 6.28 (d, *J* = 2.4 Hz, 1H), 6.12 (s, 1H), 5.99 (d, *J* = 2.4 Hz, 1H), 4.03 (d, *J* = 6.9 Hz, 2H), 2.51 (s, 3H), 2.39 (s, 3H), 2.09 (s, 3H), 1.82 (s, 3H), 1.12 (t, *J* = 7.1 Hz, 3H). ^13^C NMR (75 MHz, DMSO-*d*_*6*_) δ 196.6, 172.9, 157.4, 151.3, 147.6, 146.9, 146.1, 145.9, 145.6, 145.5, 130.4, 129.6, 129.2, 124.3, 119.6, 119.0, 117.4, 116.1, 116.0, 115.7, 115.4, 67.5, 54.7, 31.5, 24.1. MS (EI, 70 eV): m/z = 369 [M^+^]. Anal. Calcd. for C_30_H_27_N_3_O_9_S: C 59.50; H 4.49; N 6.94; Found: C 59.32; H 4.21; N 6.48.

## Supplementary Information

Supplementary Information.

## Data Availability

All data generated or analysed during this study are included in this published article and its supplementary information files.
