# Supplementary material for: Diarylation of thiazolopyrimidines by laccase and their in vitro evaluation as antitumor agents
Source: Sci Rep. 2022 Dec 25;12:22326. doi: 10.1038/s41598-022-26820-9 (PMC9790884; doi:10.1038/s41598-022-26820-9)
Supplement: Supplementary file 1 — Supplementary Information. [file 41598_2022_26820_MOESM1_ESM.docx]

**SUPPLEMENTARY INFORMATION**

**Diarylation of thiazolopyrimidines by laccase and their in vitro evaluation as antitumor agents**

Mansour Shahedi^a^, Rojina Shahani^a^, Zohreh Habibi ^*a^, Maryam Yousefi ^**b^, Jesper Brask^c^, Arash Minai-Tehrani^b^, Fatemeh Yazdi Samadi^b^ and Mehdi Mohammadi^d^

^a^ Department of Organic and Inorganic Chemistry, Faculty of Chemistry, Shahid Beheshti University, G.C., Tehran, Iran

^b^ Nanobiotechnology Research Center, Avicenna Research Institute, ACECR, Tehran, Iran

^c^ Novozymes A/S, Krogshøjvej 36, 2880 Bagsværd, Copenhagen, Denmark.

^d^ Bioprocess Engineering Department, Institute of Industrial and Environmental Biotechnology, National Institute of Genetic Engineering and Biotechnology (NIGEB), Tehran, Iran.

^*^ Corresponding author. Tel.:+982 129 903 110; fax: +982 122 431 663.E-mail addresses: Z_habibi@sbu.ac.ir (Z. Habibi)

^**^ Corresponding author. Tel.:+982 122 432 020; fax: +982 122 432 021. E-mail addresses: M.yousefi@ari.ir (M. Yousefi)

CONTENTS

Copies of HMBC, HSQC, H,H-COSY, DEPT-135, ^1^H NMR, ^13^C NMR and Mass spectra of the products ----------------S4

**Figure Legends:**

Figure 1S. Compound **2c** ^1^H NMR

Figure 2S. Compound **2c** ^13^C NMR

Figure 3S. Compound **2f** ^13^H NMR

Figure 4S. Compound **2f** ^13^C NMR

Figure 5S. Compound **4a** ^1^H NMR

Figure 6S. Compound **4a** ^13^C NMR

Figure 7S. Compound **4a** ^13^C NMR and DEPT-135

Figure 8S. Compound **4a** ^13^C NMR and DEPT-135

Figure 9S. Compound **4a** ^13^C NMR and DEPT-135

Figure 10S. Compound **4a** H, H-COSY

Figure 11S. Compound **4a** H, H-COSY

Figure 12S. Compound **4a** HMBC

Figure 13S. Compound **4a** HMBC

Figure 14S. Compound **4a** HSQC

Figure 15S. Compound **4a** Mass spectra

Figure 16S. Compound **4b** ^1^H NMR

Figure 17S. Compound **4b**^13^C NMR

Figure 18S. Compound **4b** Mass spectra

Figure 19S. Compound **4c** ^1^H NMR

Figure 20S. Compound **4c** ^13^C NMR

Figure 21S.Compound **4c** Mass spectra

Figure 22S.Compound **4d** ^1^H NMR

Figure 23S.Compound **4d** ^13^C NMR

Figure 24S. Compound **4d** Mass spectra

Figure 25S. Compound **4e** ^1^H NMR

Figure 26S. Compound **4e** ^13^C NMR

Figure 27S. Compound **4e** Mass spectra

Figure 28S. Compound **4f** ^1^H NMR (in acetone-d*_6_*)

Figure 29S. Compound **4f** ^1^H NMR (in DMSO-*d_6_)*

Figure 30S. Compound **4f** ^13^C NMR

Figure 31S. Compound **4f** Mass spectra

Figure 32S. Compound **4g** ^1^H NMR (in acetone-d*_6_*)

Figure 33S. Compound **4g** ^1^H NMR (in DMSO-*d_6_)*

Figure 34S. Compound **4g** ^13^C NMR

Figure 35S. Compound **4g** Mass spectra

Figure 36S. Compound **4h** ^1^H NMR (in acetone-d*_6_*)

Figure 37S. Compound **4h** ^1^H NMR (in DMSO-*d_6_)*

Figure 38S. Compound **4h** ^13^C NMR

Figure 39S. Compound **4h** Mass spectra

Figure 40S. Compound **4i** ^1^H NMR

Figure 41S. Compound **4i** ^13^C NMR

Figure 42S. Compound **4i** Mass spectra

Figure 43S. Compound **4j** ^1^H NMR

Figure 44S. Compound **4j** ^13^C NMR

Figure 45S. Compound **4j** Mass spectra

Figure 46S. Compound **4k** ^1^H NMR

Figure 47S. Compound **4k** ^13^C NMR

Figure 48S. Compound **4k** Mass spectra

Figure 49S. Compound **4n** ^1^H NMR

Figure 50S. Compound **4n** ^13^C NMR

Figure 51S Compound **4n** Mass spectra

Figure 52S. Compound **4o** ^1^H NMR

Figure 53S. Compound **4o** ^13^C NMR

Figure 54S Compound **4o** Mass spectra

**Copies of HMBC, HSQC, H,H-COSY, DEPT-135, ^1^H NMR, ^13^C NMR and Mass spectra of the products**


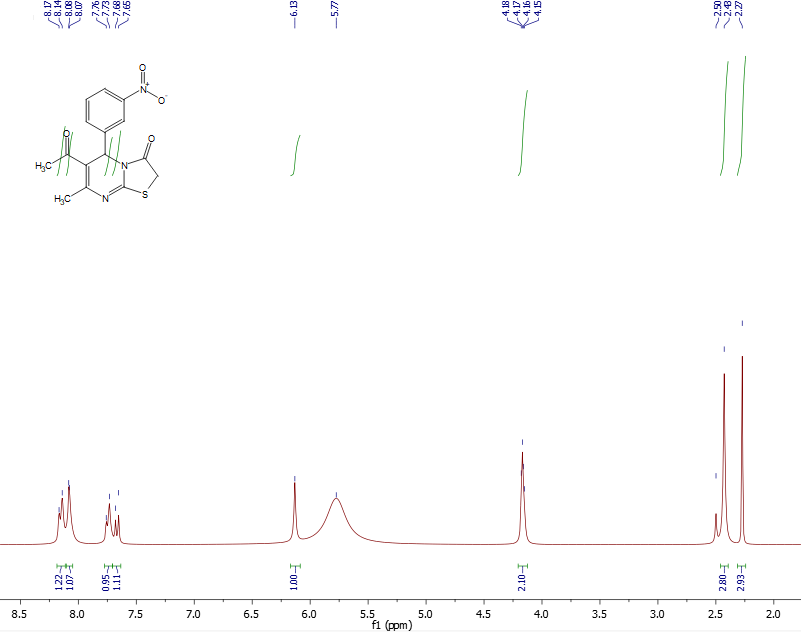


**Figure 1S** ^1^H NMR (300 MHz, DMSO-*d_6_*) of compound **2c**


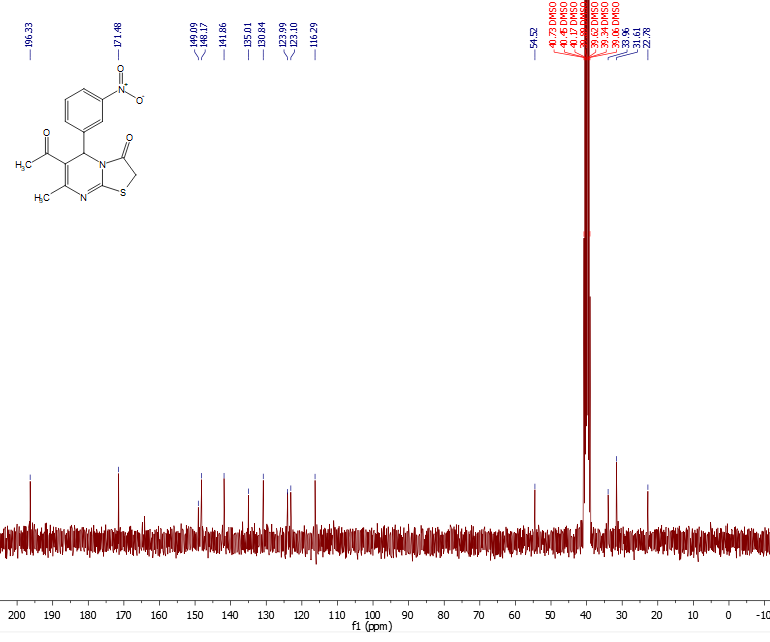


**Figure 2S** ^13^C NMR (75 MHz, DMSO-*d_6_*) of compound **2c**


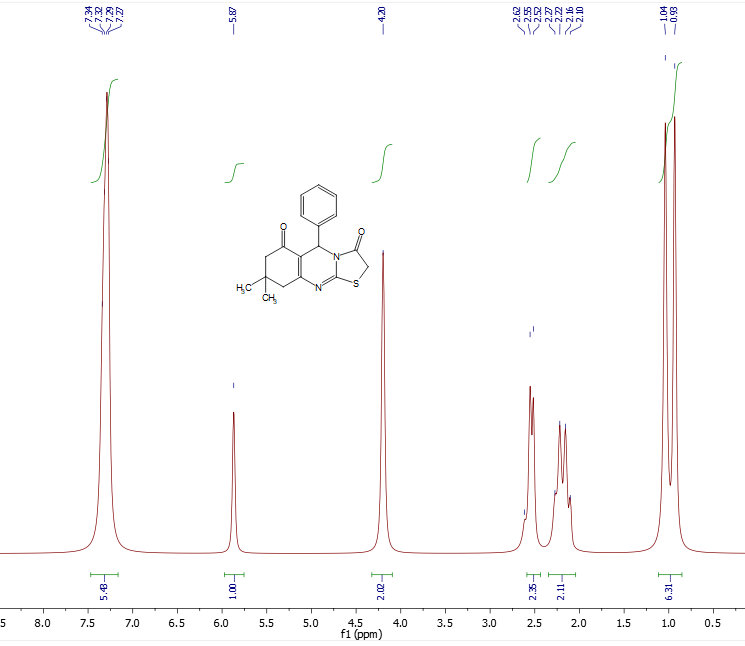


**Figure 3S** ^1^H NMR (300 MHz, DMSO-*d_6_*) of compound **2f**


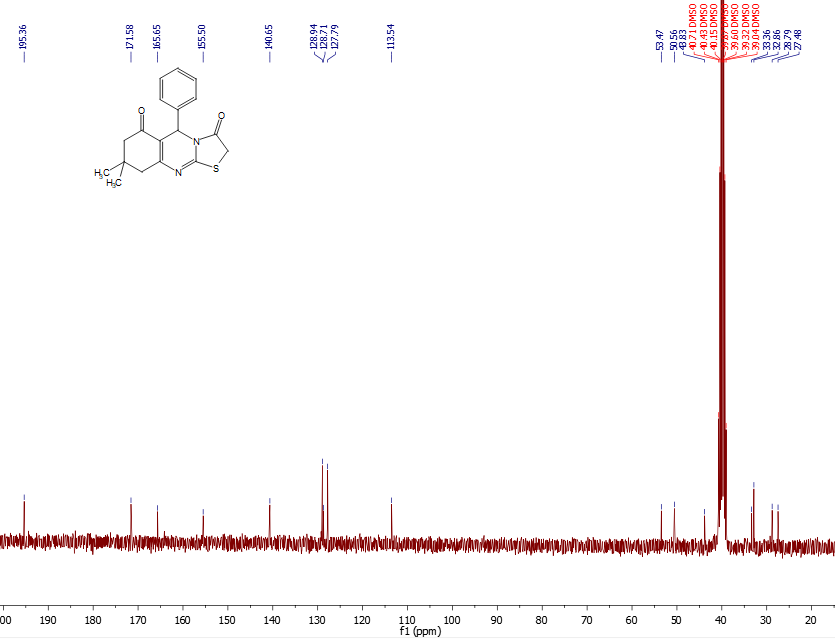


**Figure 4S** ^13^C NMR (75 MHz, DMSO-*d_6_*) of compound **2f**


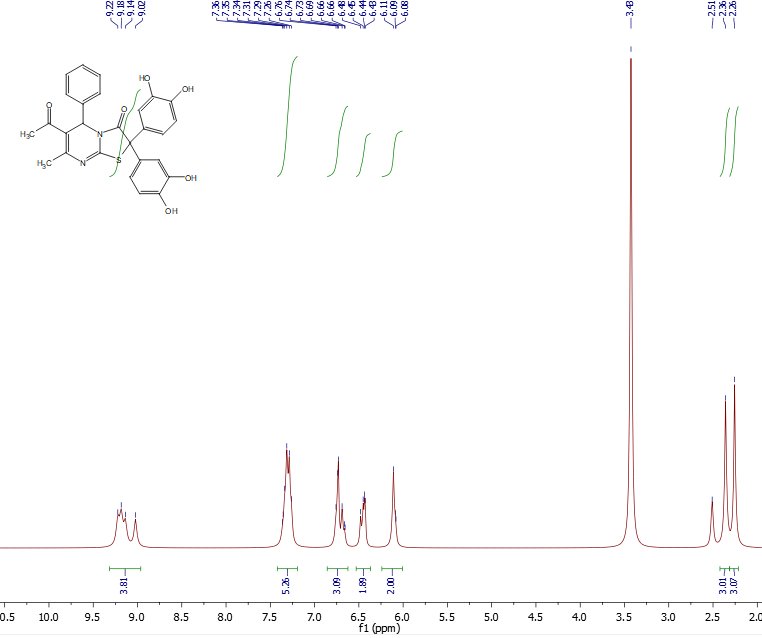


**Figure 5S.** ^1^H NMR (300 MHz, DMSO-*d_6_*) of compound **4a**


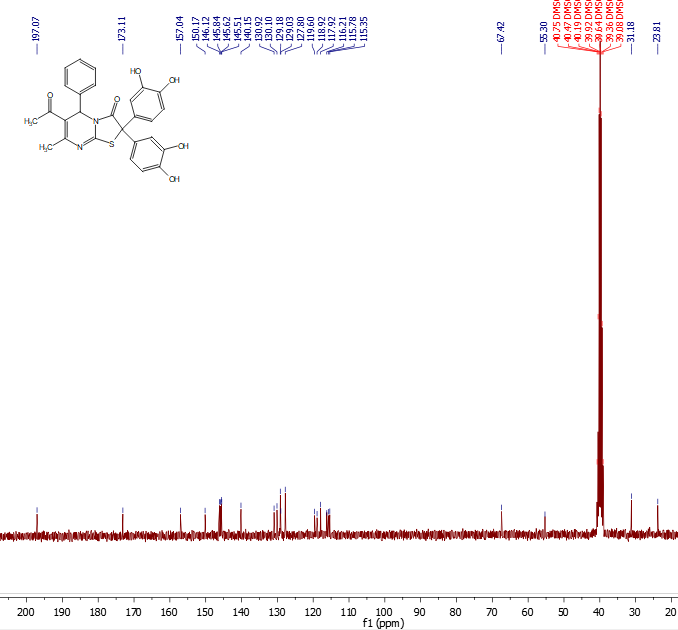


**Figure 6S.** ^13^CNMR (75 MHz, DMSO-*d_6_*) of compound **4a**


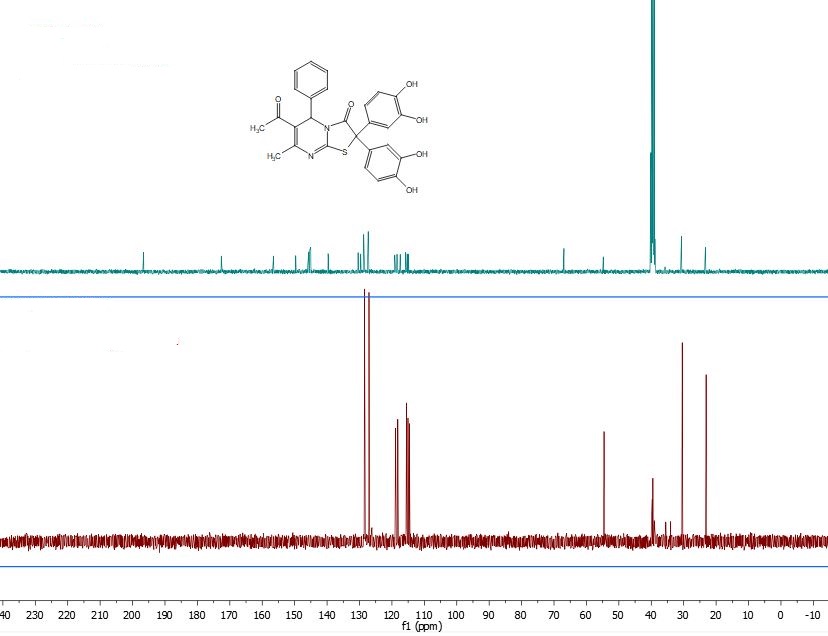


Dept-135

^13^CNMR

**Figure 7S.** ^13^C NMR and DEPT-135 (100 MHz, DMSO-*d_6_*) of compound **4a**


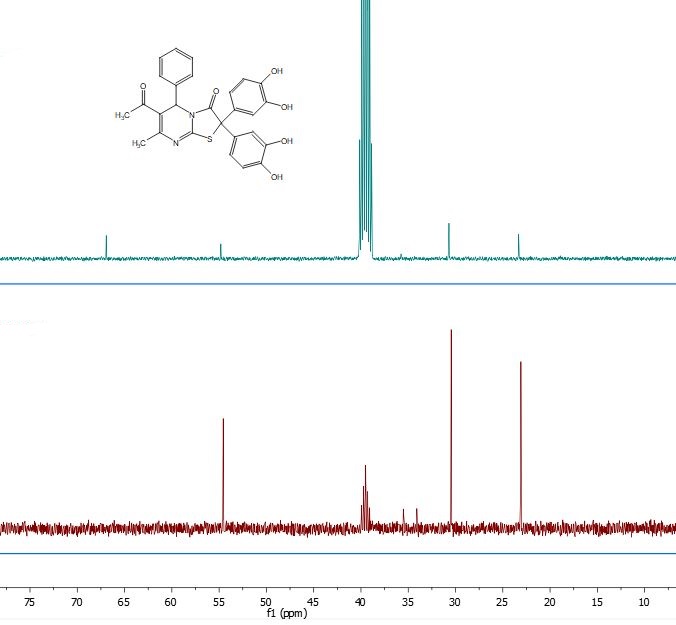


^13^CNMR

Dept-135

**Figure 8S.** ^13^C NMR and DEPT-135 (100 MHz, DMSO-*d_6_*) of compound **4a**


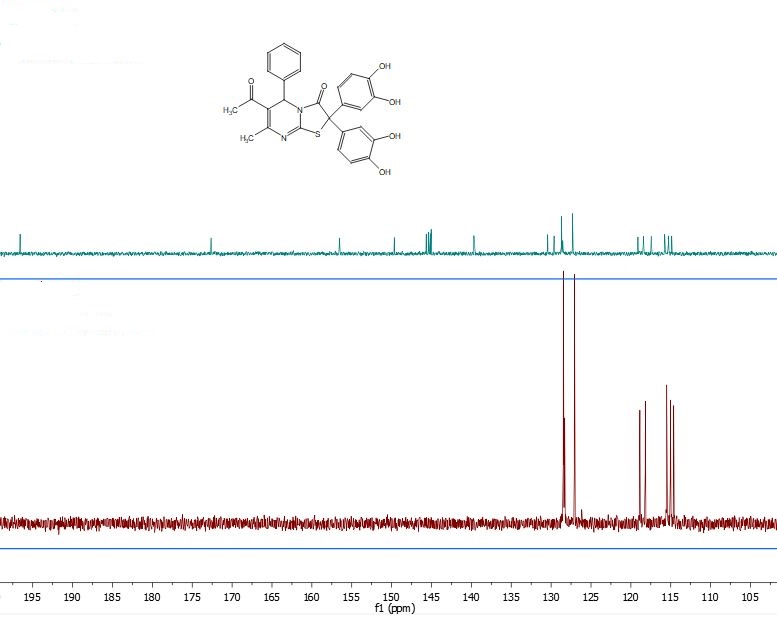


Dept-135

^13^CNMR

**Figure 9S.** ^13^C NMR and DEPT-135 (100 MHz, DMSO-*d_6_*) of compound **4a**


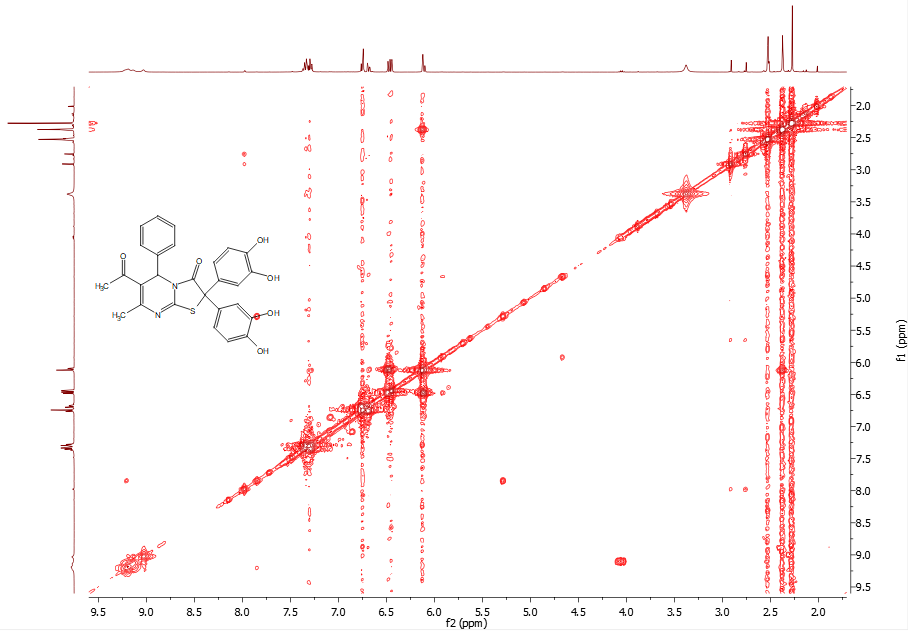


**Figure 10S.** H, H-COSY (400 MHz, DMSO-*d_6_*) of compound **4a**


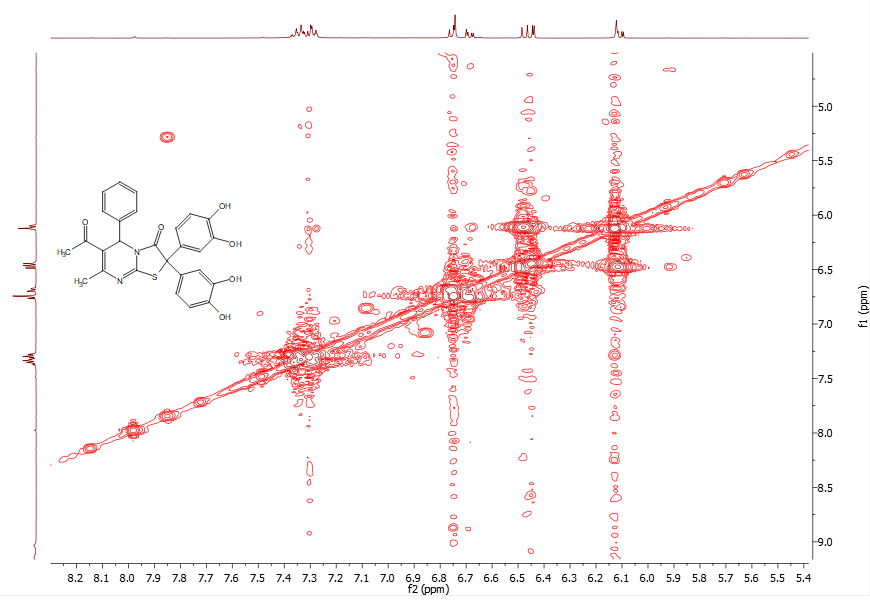


**Figure 11S.** H, H-COSY (400 MHz, DMSO-*d_6_*) of compound **4a**


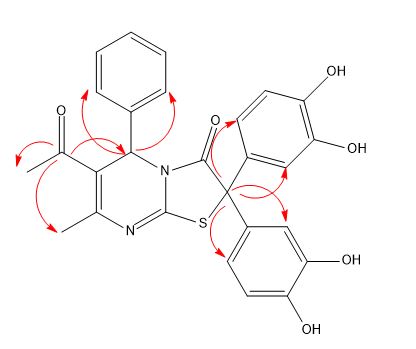

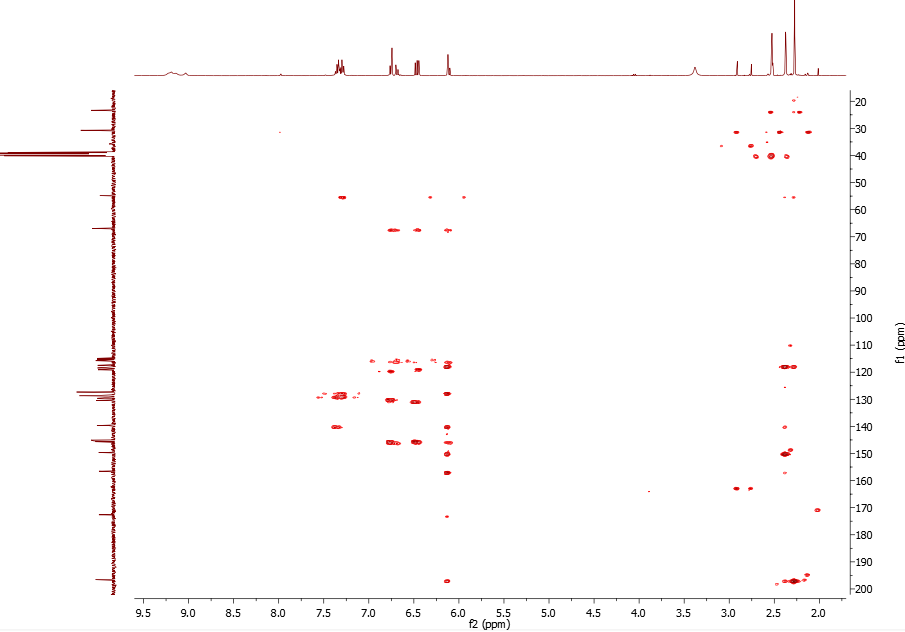


**Figure 12S.** HMBC (400 MHz, DMSO-*d_6_*) of compound **4a**


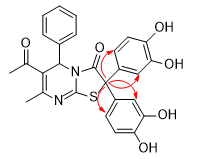


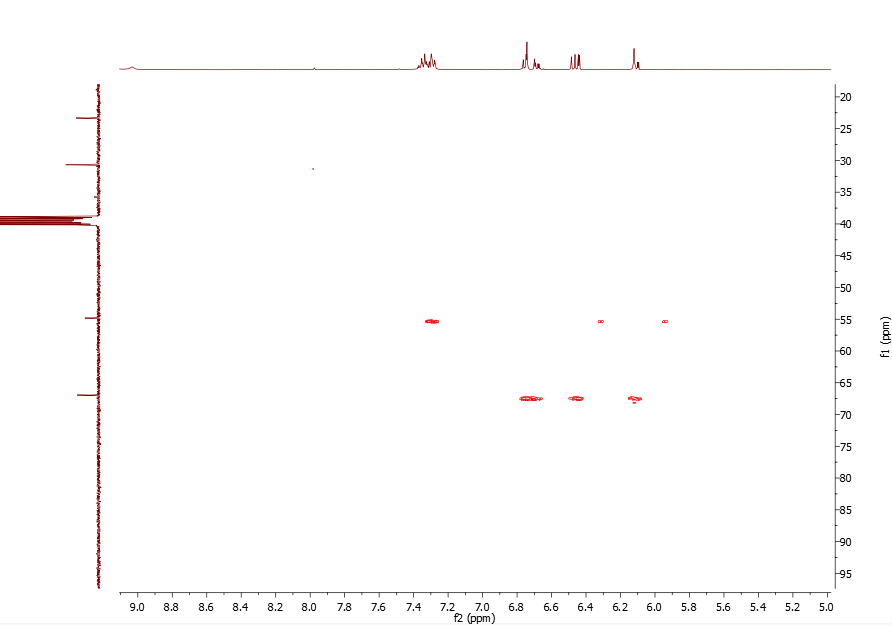


**Figure 13S.** HMBC (400 MHz, DMSO-*d_6_*) of compound **4a**


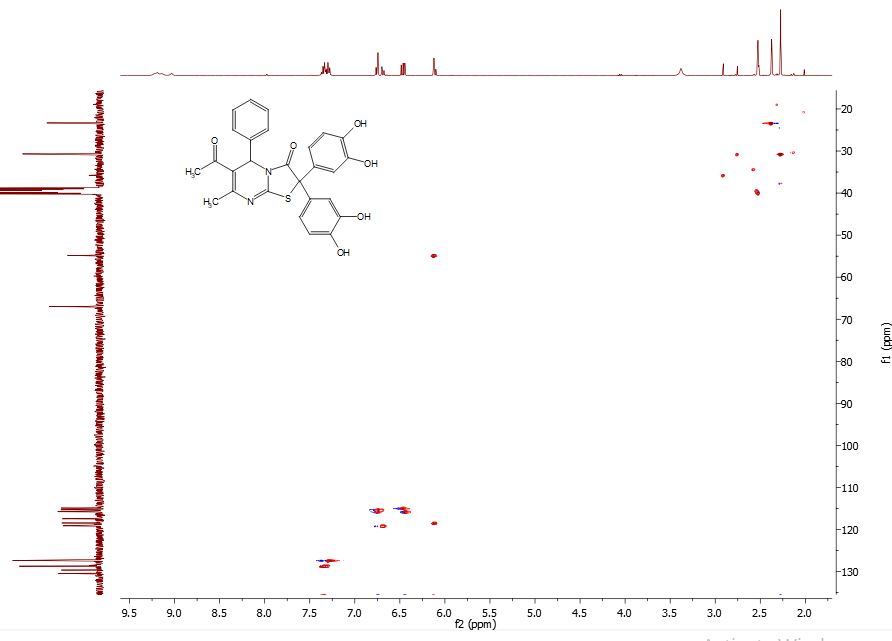


**Figure 14S.** HSQC (400 MHz, DMSO-*d_6_*) of compound **4a**


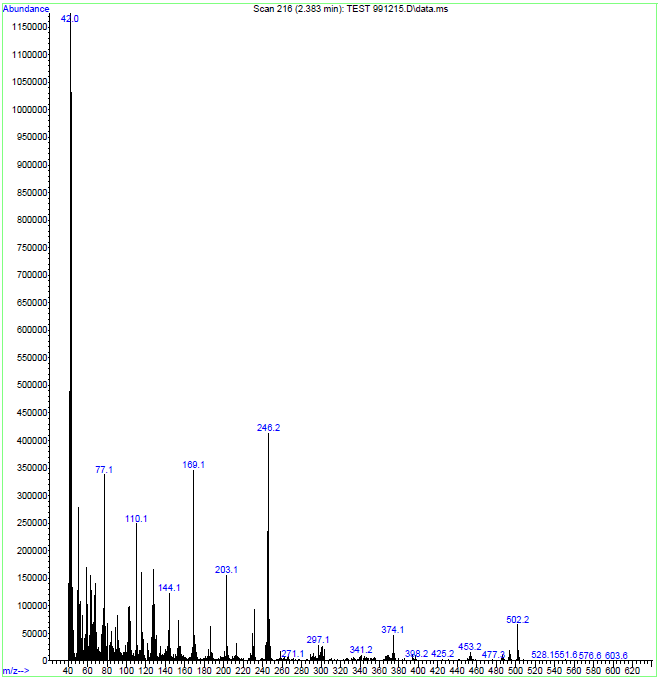

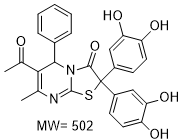


**Figure 15S.** Mass spectra of **4a**


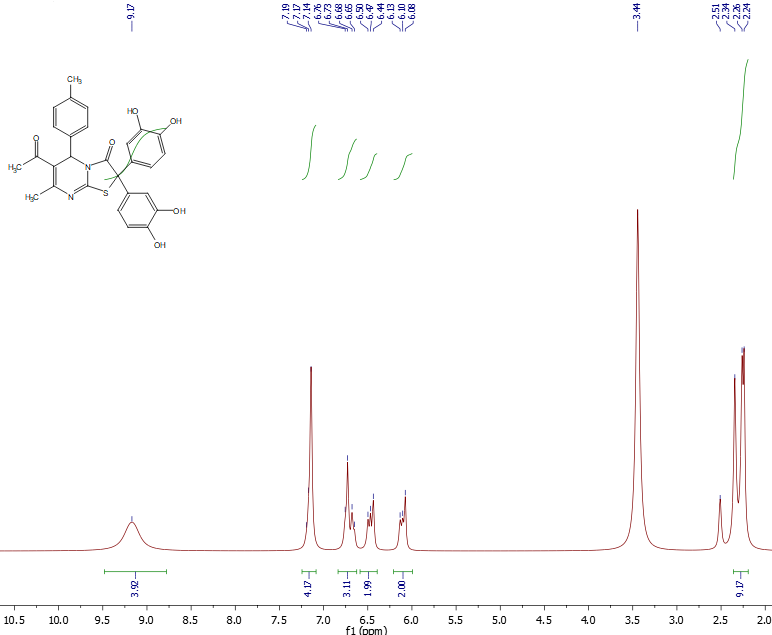


**Figure 16S.** ^1^HNMR (300 MHz, DMSO-*d_6_*) of compound **4b**


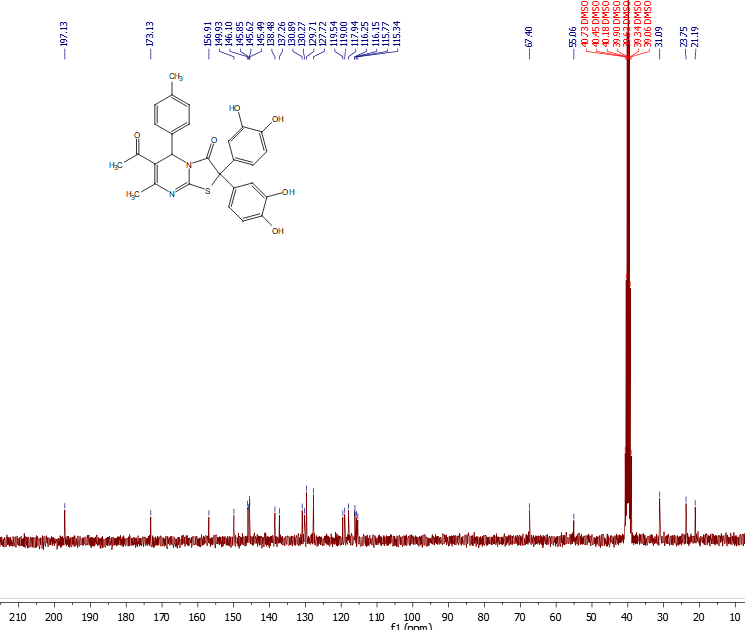


**Figure 17S.** ^13^CNMR (75 MHz, DMSO-*d_6_*) of compound **4b**


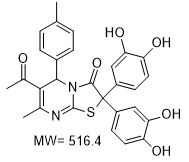


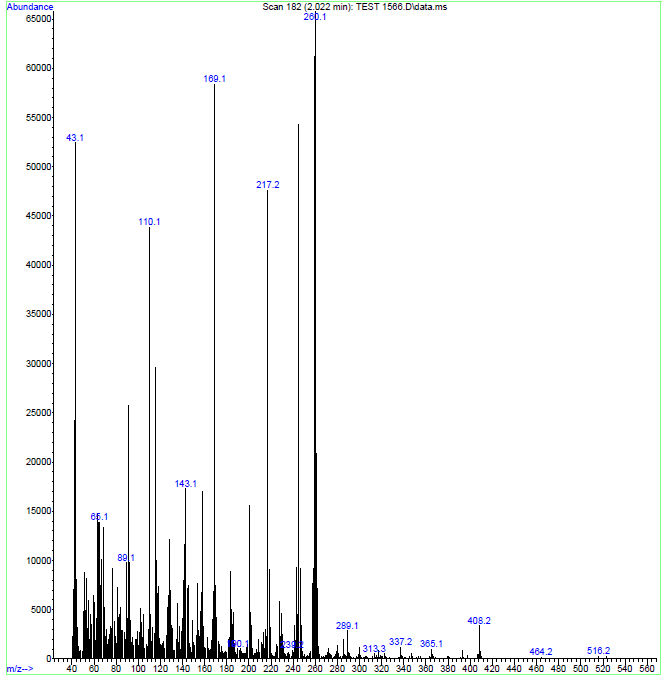


**Figure 18S.** Mass spectra of **4b**


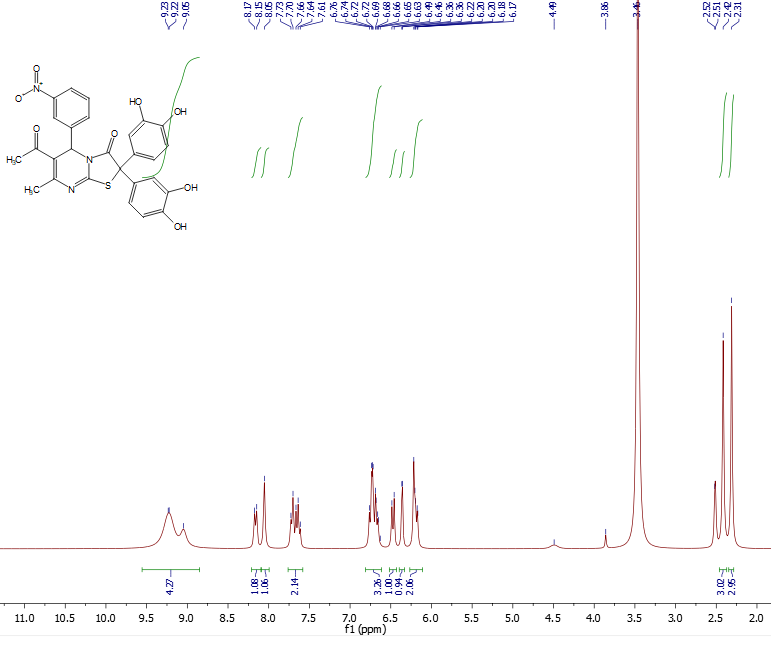


**Figure 19S.** ^1^HNMR (300 MHz, DMSO-*d_6_*) of compound **4c**


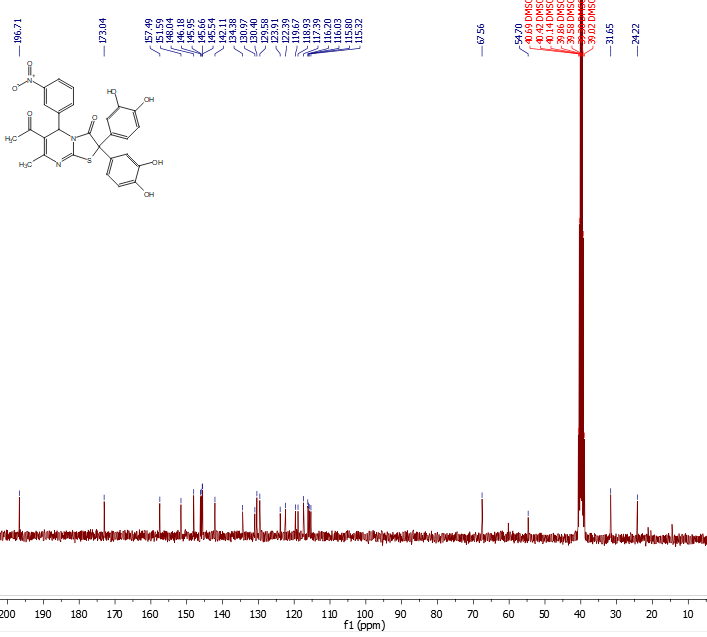


**Figure 20S.** ^13^CNMR (75 MHz, DMSO-*d_6_*) of compound **4c**

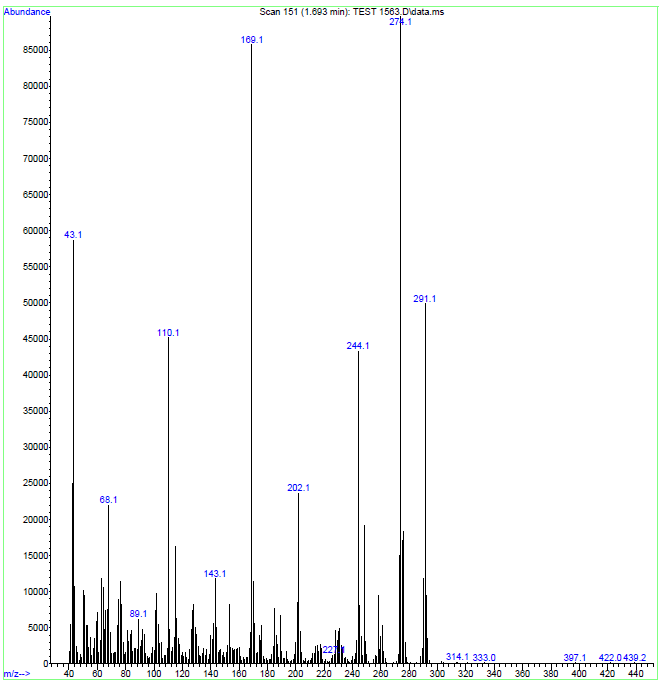


**Figure 21S**. Mass spectra of **4c**


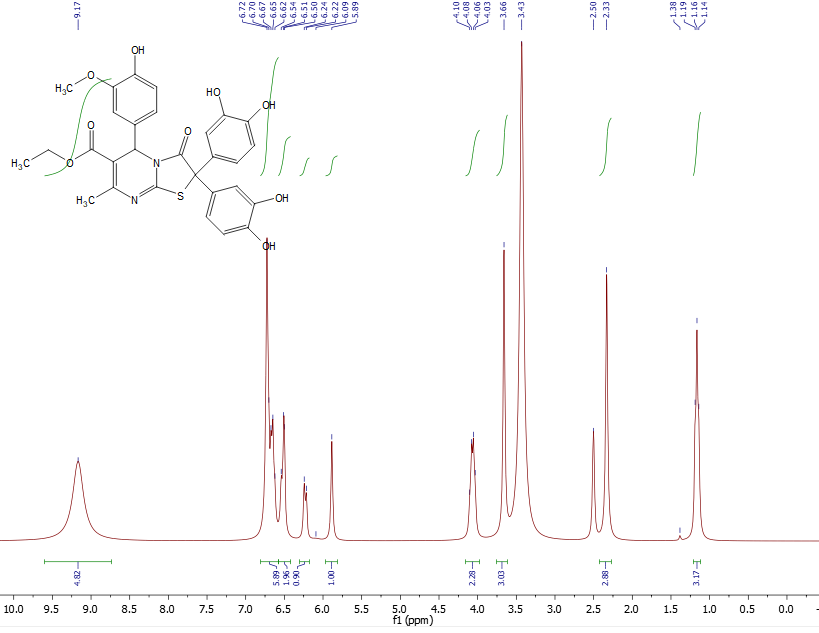


**Figure 22S.** ^1^HNMR (300 MHz, DMSO-*d_6_*) of compound **4d**


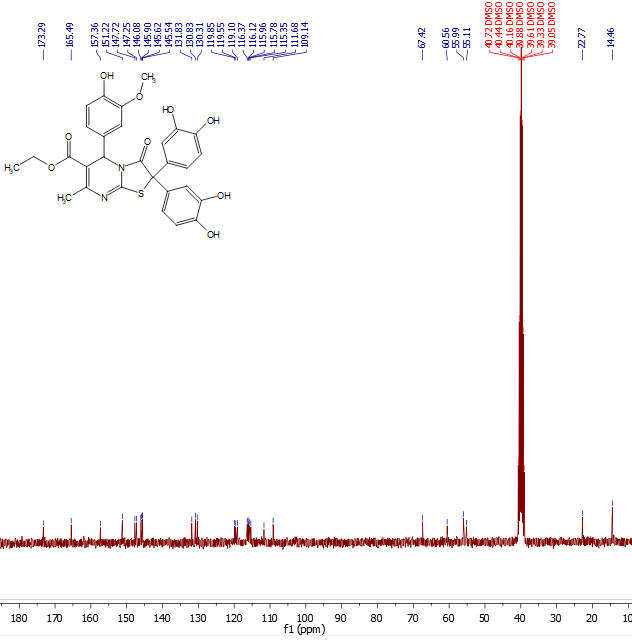


**Figure 23S.** ^13^CNMR (75 MHz, DMSO-*d_6_*) of compound **4d**

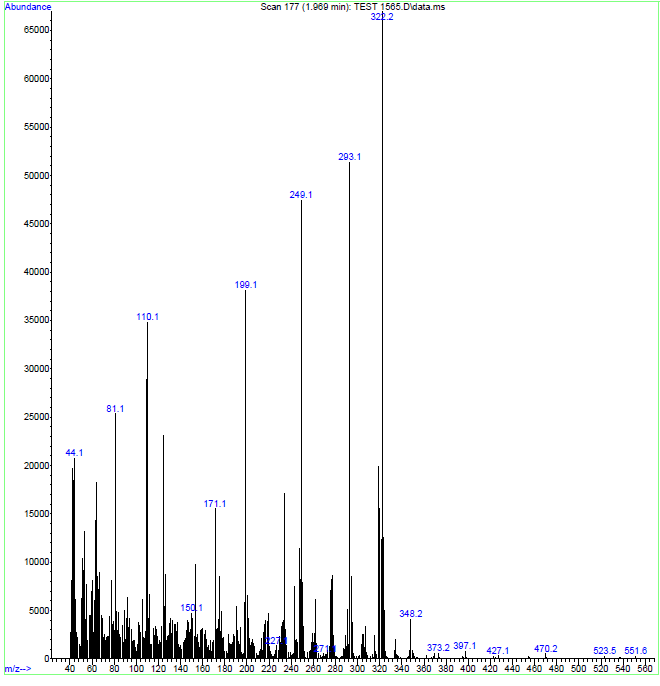


**Figure 24S**. Mass spectra of **4d**


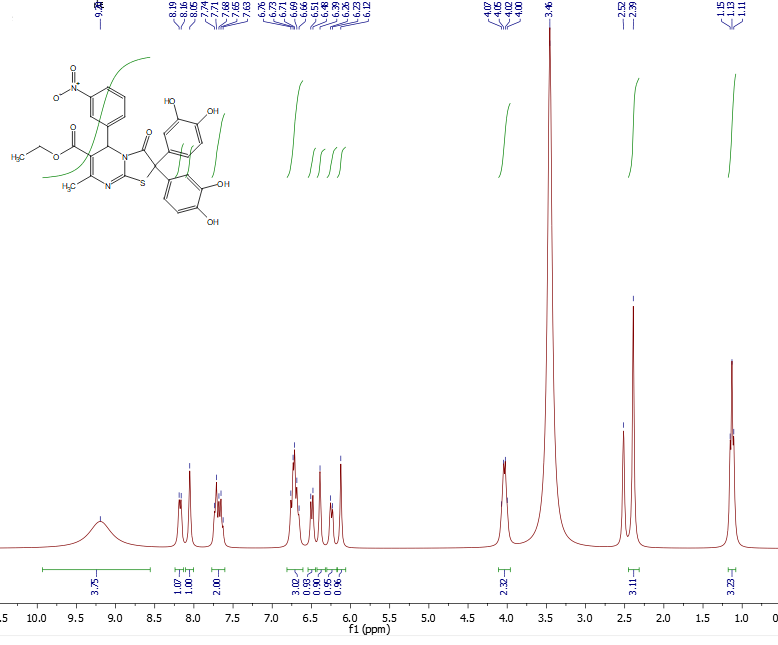


**Figure 25S.** ^1^HNMR (300 MHz, DMSO-*d_6_*) of compound **4e**


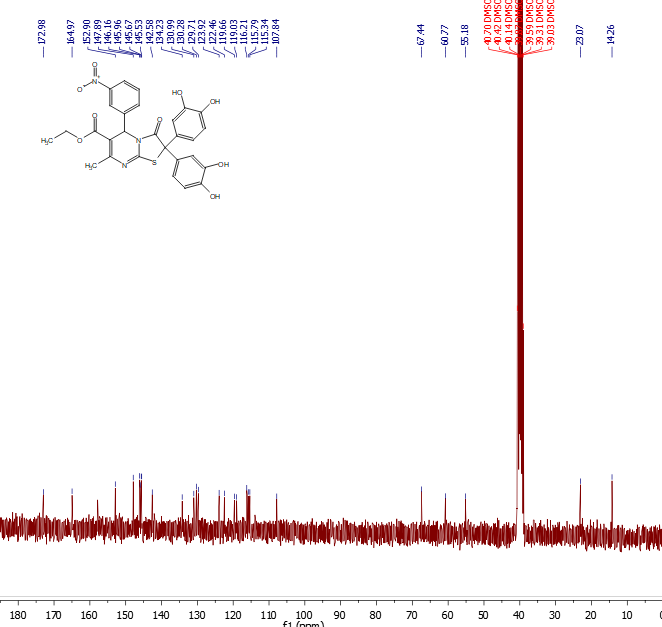


**Figure 26S.** ^13^CNMR (75 MHz, DMSO-*d_6_*) of compound **4e**

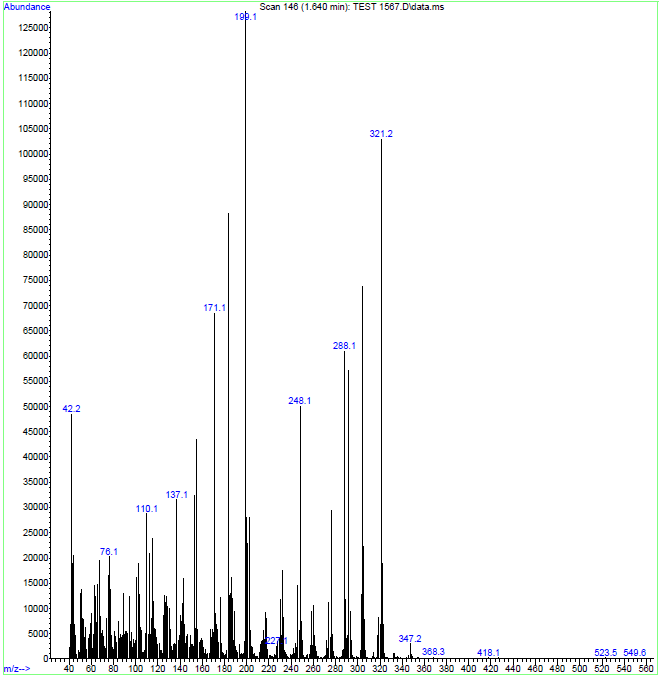


**Figure 27S**. Mass spectra of **4e**


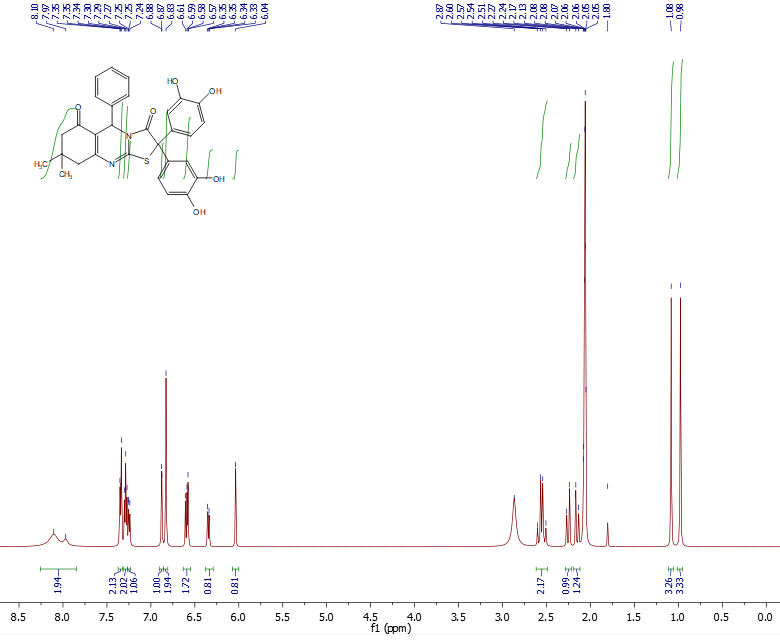


**Figure 28S.** ^1^HNMR (300 MHz, acetone-d*_6_*) of compound **4f**


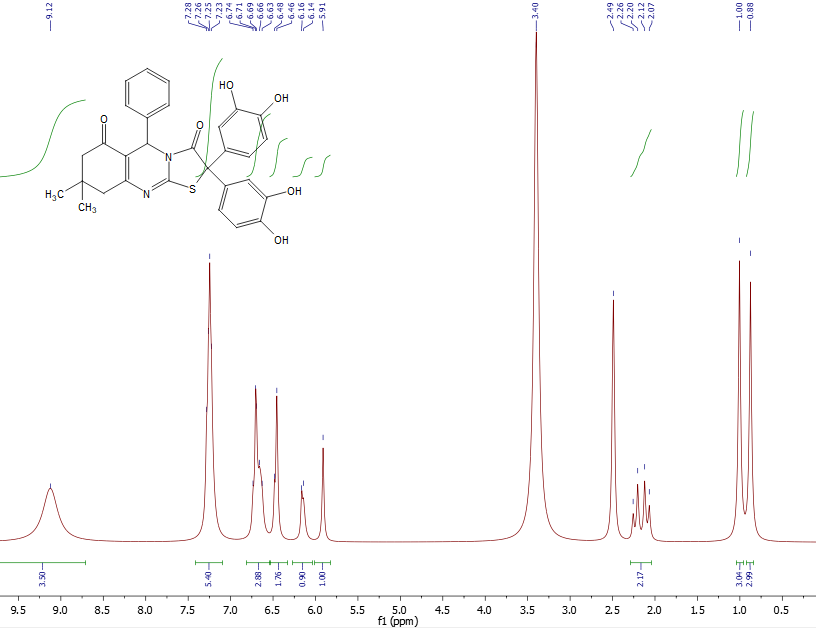


**Figure 29S.** ^1^HNMR (300 MHz, DMSO-*d_6_*) of compound **4f**


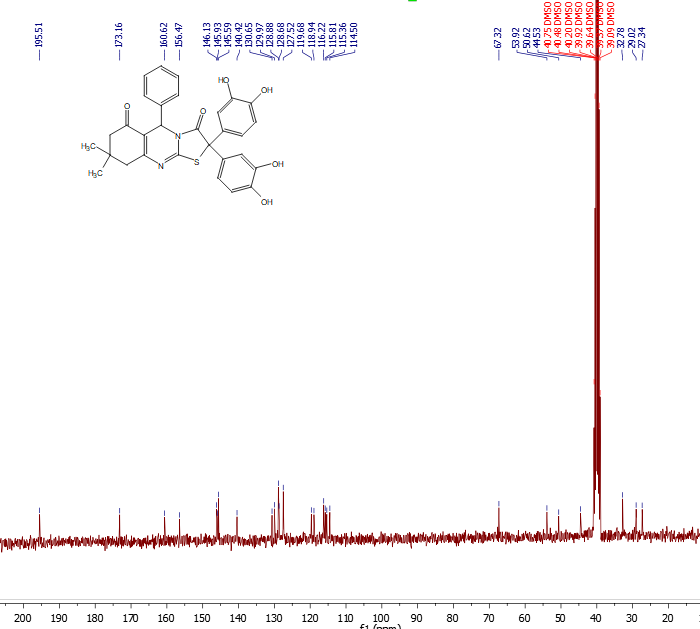


**Figure 30S.** ^13^CNMR (75 MHz, DMSO-*d_6_*) of compound **4f**

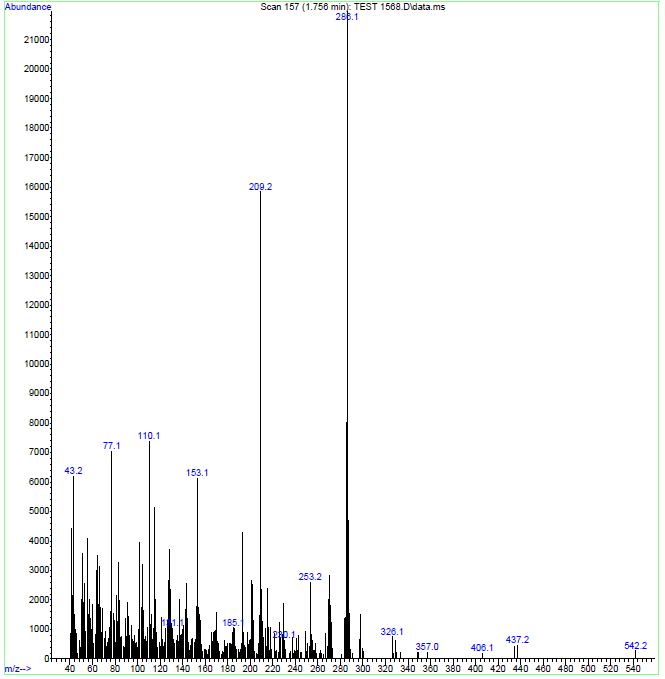


**Figure 31S** Mass spectra of **4f**


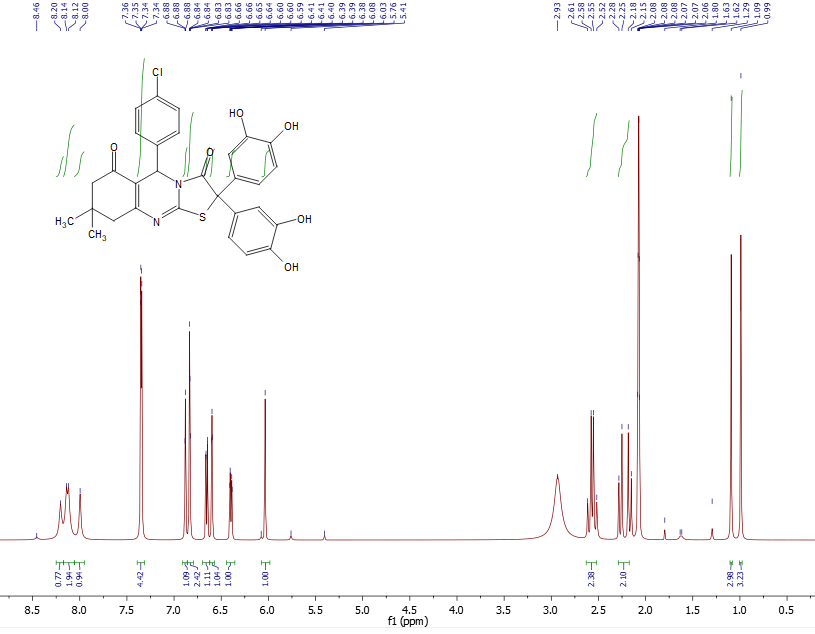


**Figure 32S.** ^1^HNMR (300 MHz, acetone-d*_6_*) of compound **4g**


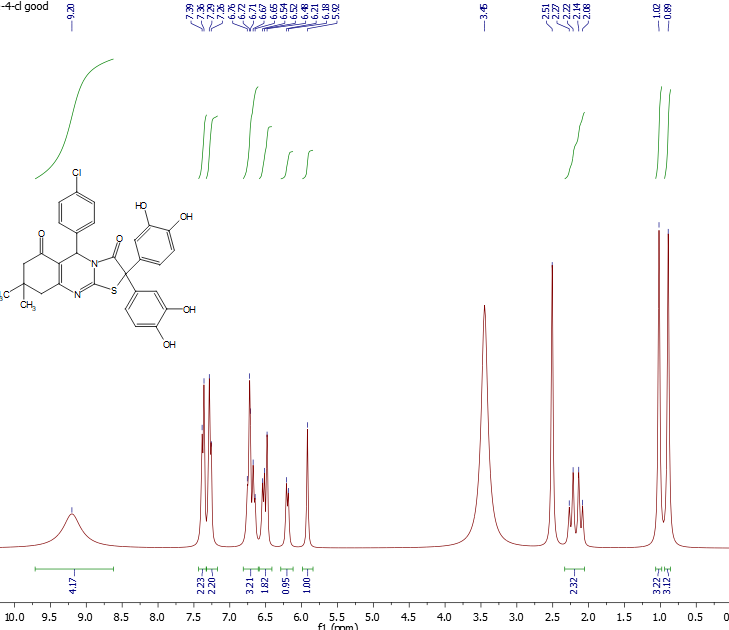


**Figure 33S.** ^1^HNMR (300 MHz, DMSO -*d_6_*) of compound **4g**


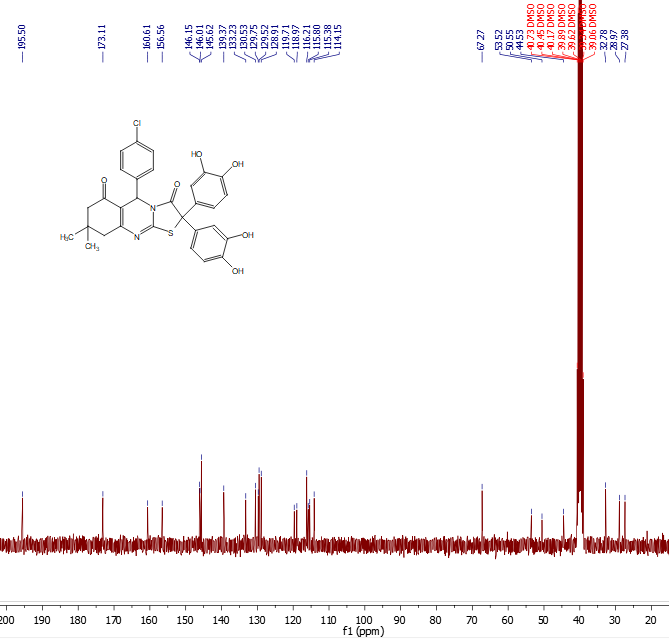


**Figure 34S.** ^13^CNMR (75 MHz, DMSO-*d_6_*) of compound **4g**

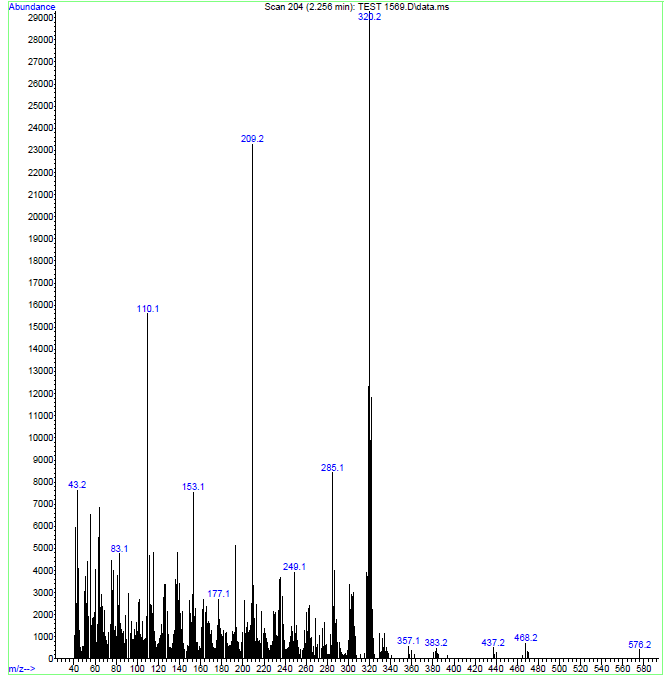


**Figure 35S**. Mass spectra of **4g**


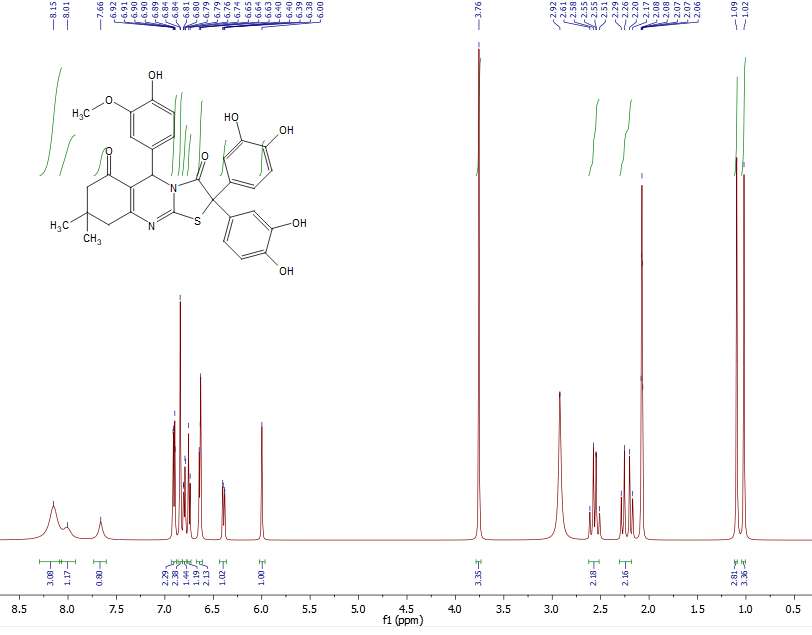


**Figure 36S.** ^1^HNMR (300 MHz, acetone-d*_6_*) of compound **4h**


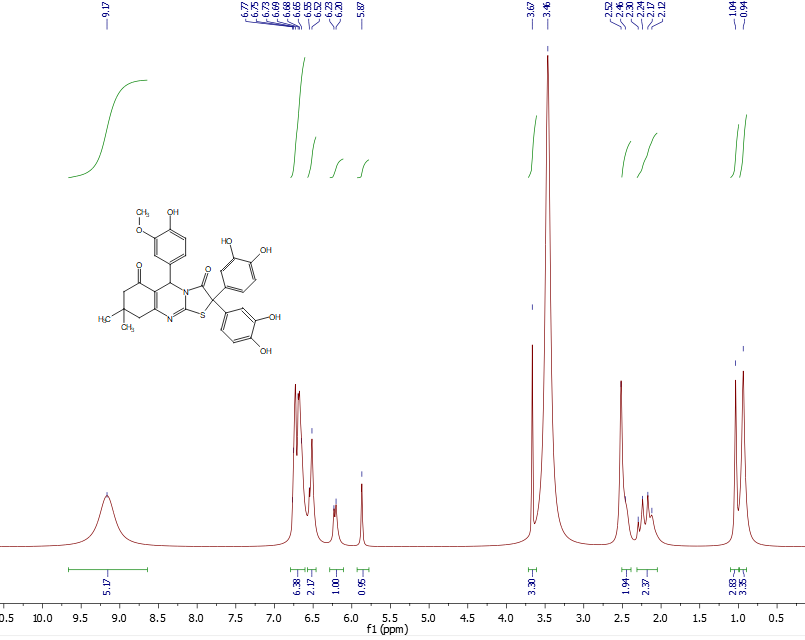


**Figure 37S.** ^1^HNMR (300 MHz, DMSO-*d_6_*) of compound **4h**


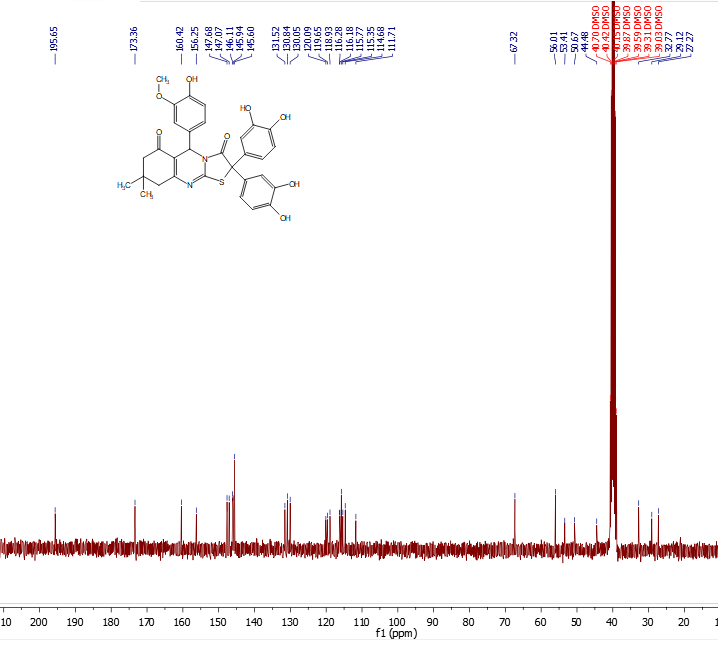


**Figure 38S.** ^13^CNMR (75 MHz, DMSO-*d_6_*) of compound **4h**

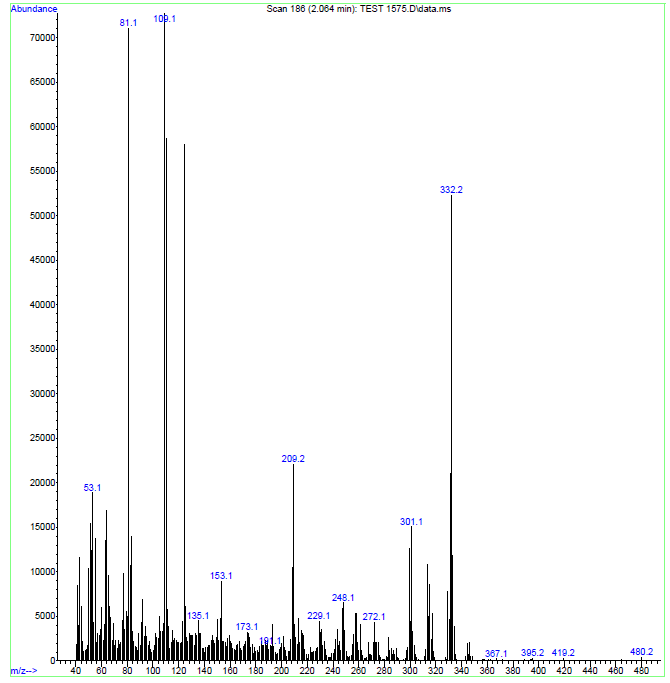


**Figure 39S.** Mass spectra of **4h**


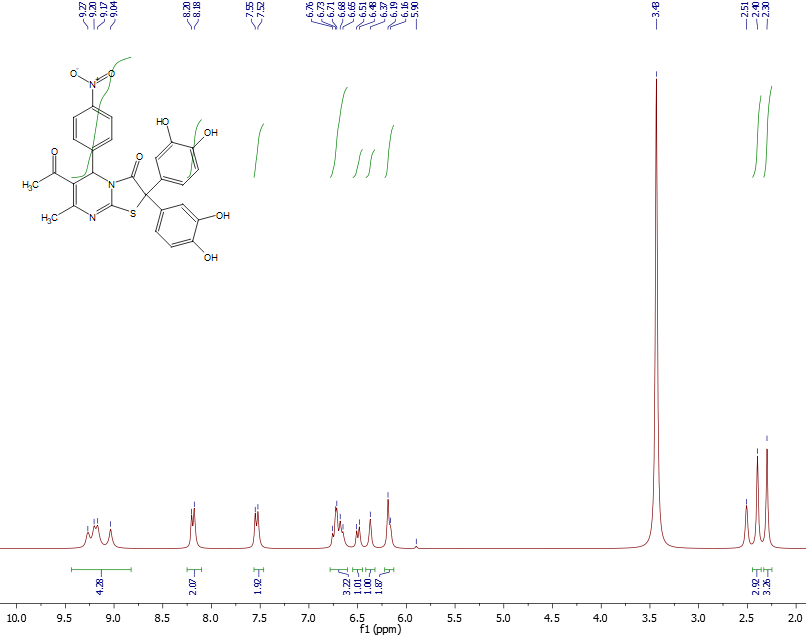


**Figure 40S.** ^1^HNMR (300 MHz, DMSO -*d_6_*) of compound **4i**


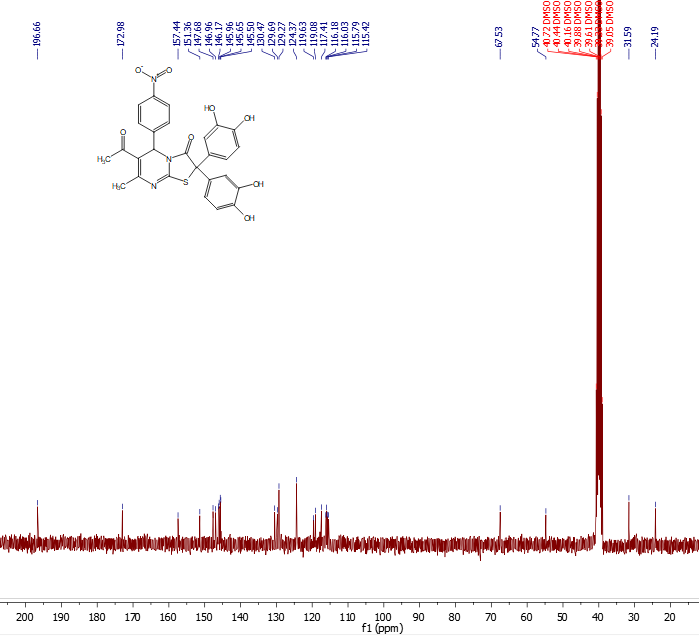


**Figure 41S.** ^13^CNMR (75 MHz, DMSO-*d_6_*) of compound **4i**

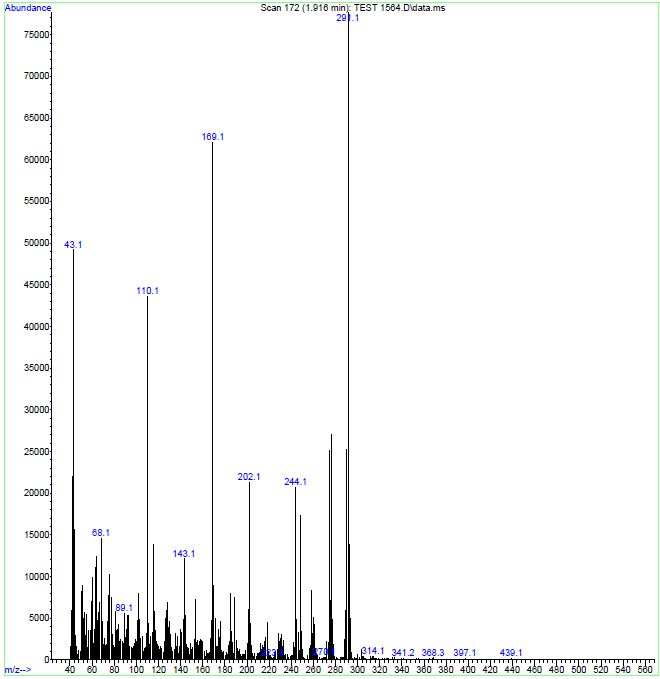


**Figure 42S.** Mass spectra of **4i**


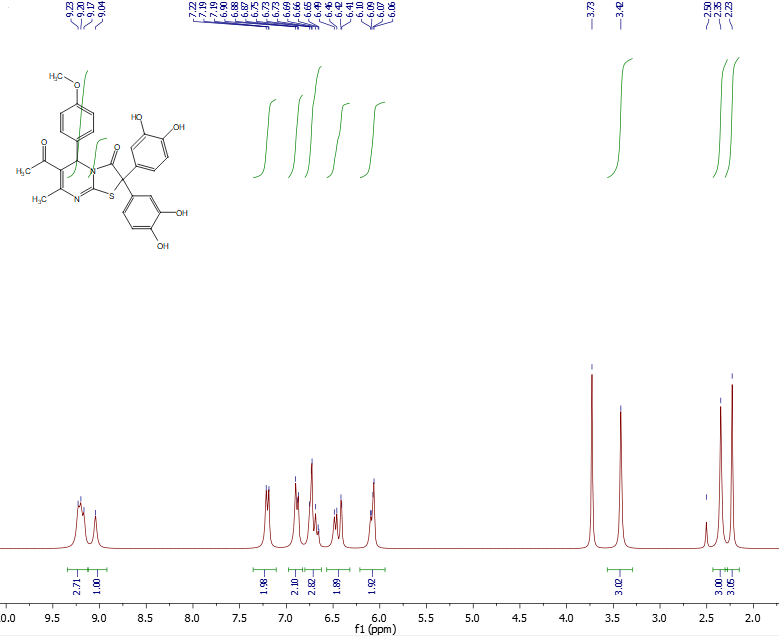


**Figure 43S.** ^1^HNMR (300 MHz, DMSO -*d_6_*) of compound **4j**


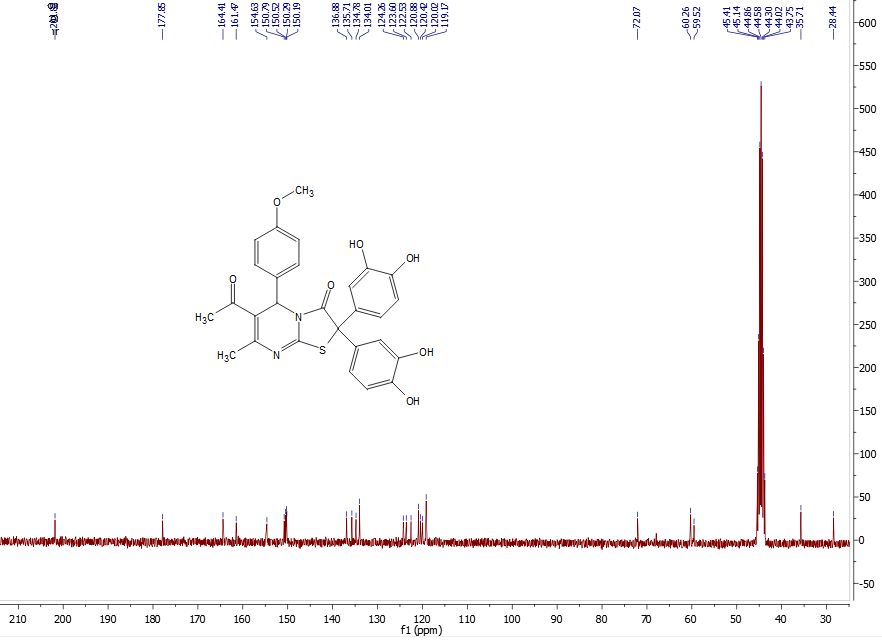


**Figure 44S.** ^13^CNMR (75 MHz, DMSO -_d_*_6_*) of compound **4j**

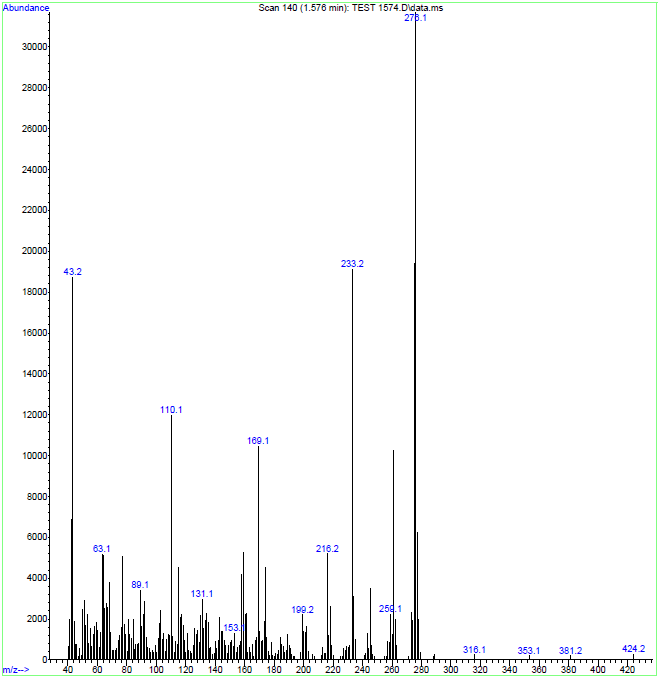


**Figure 45S.** Mass spectra of **4j**


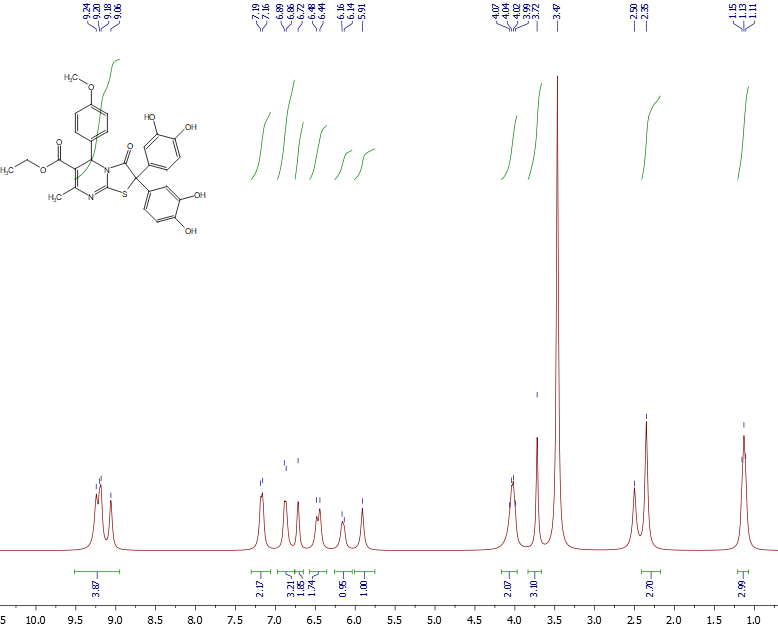


**Figure 46S.** ^1^HNMR (300 MHz, DMSO -*d_6_*) of compound **4k**


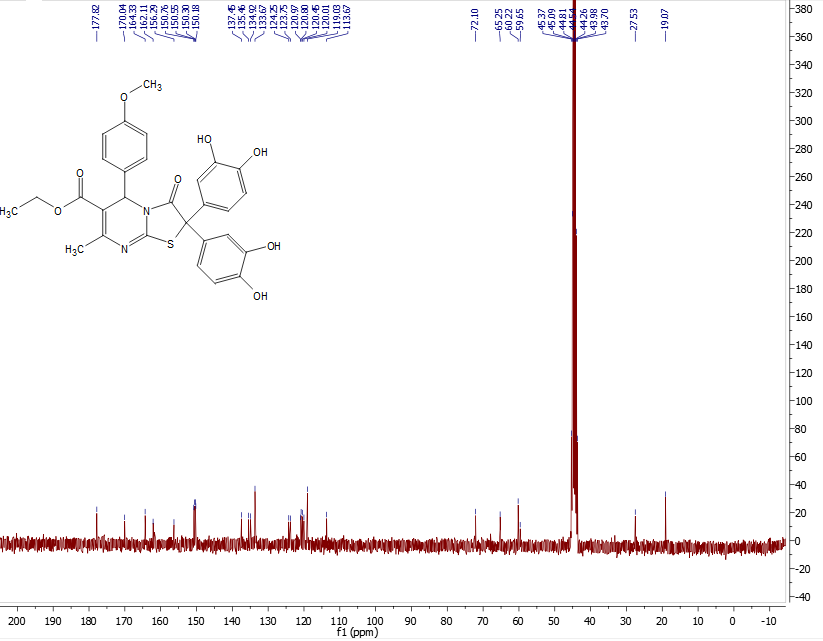


**Figure 47S.** ^13^CNMR (75 MHz, DMSO -*d_6_*) of compound **4k**

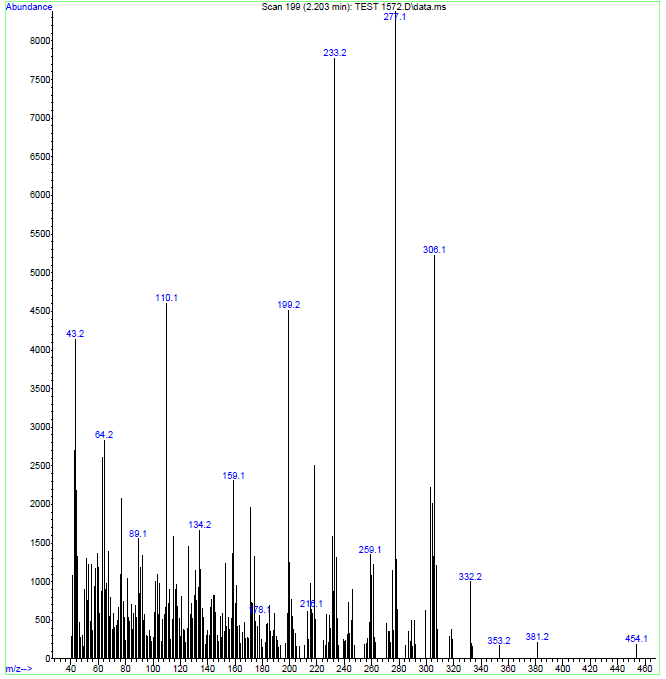


**Figure 48S.** Mass spectra of **4k**


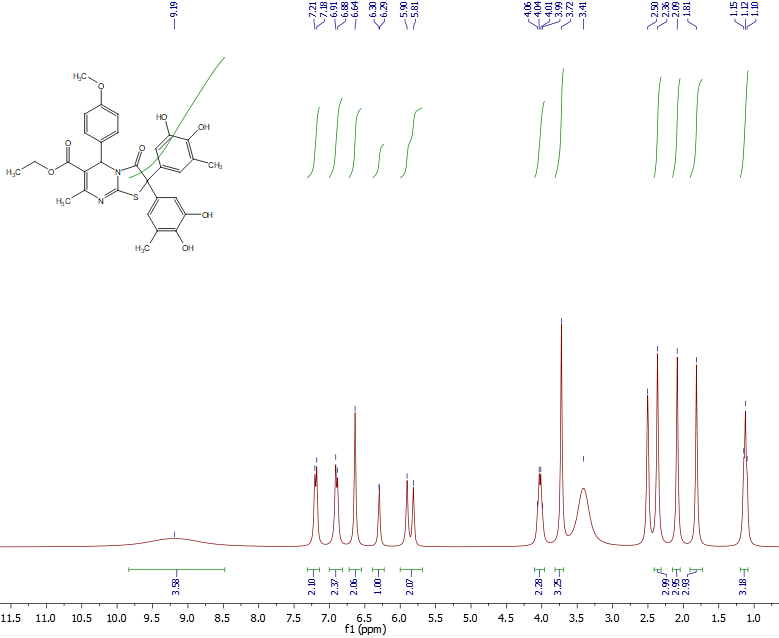


**Figure 49S.** ^1^HNMR (300 MHz, DMSO -*d_6_*) of compound **4n**


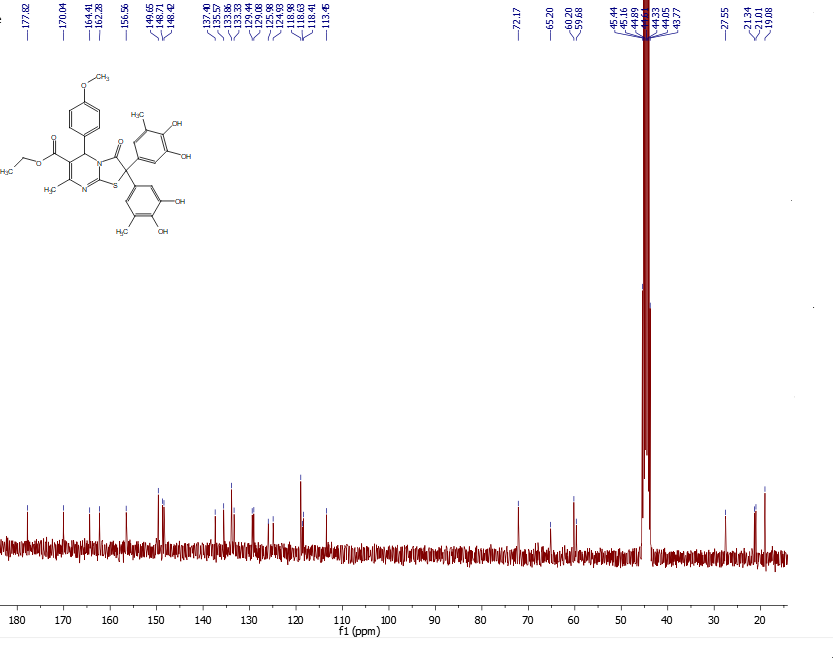


**Figure 50S.** ^13^CNMR (75 MHz, DMSO -*d_6_*) of compound **4n**

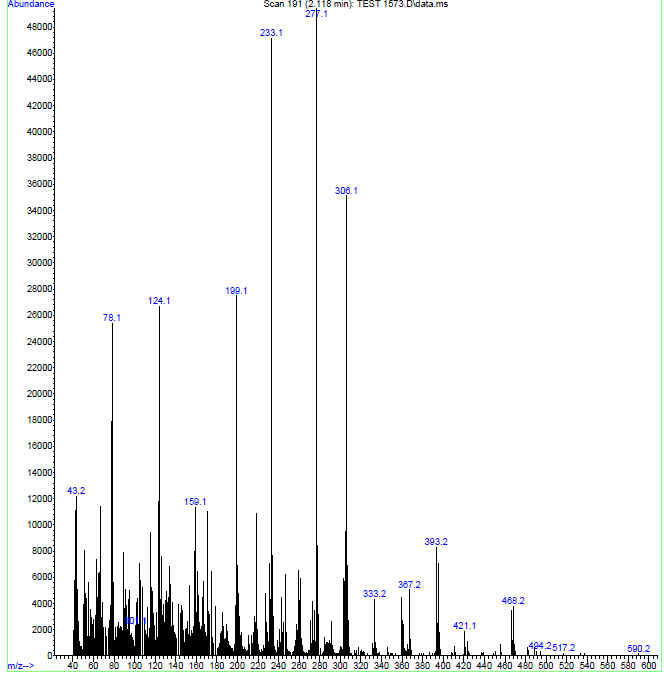


**Figure 51S.** Mass spectra of **4n**


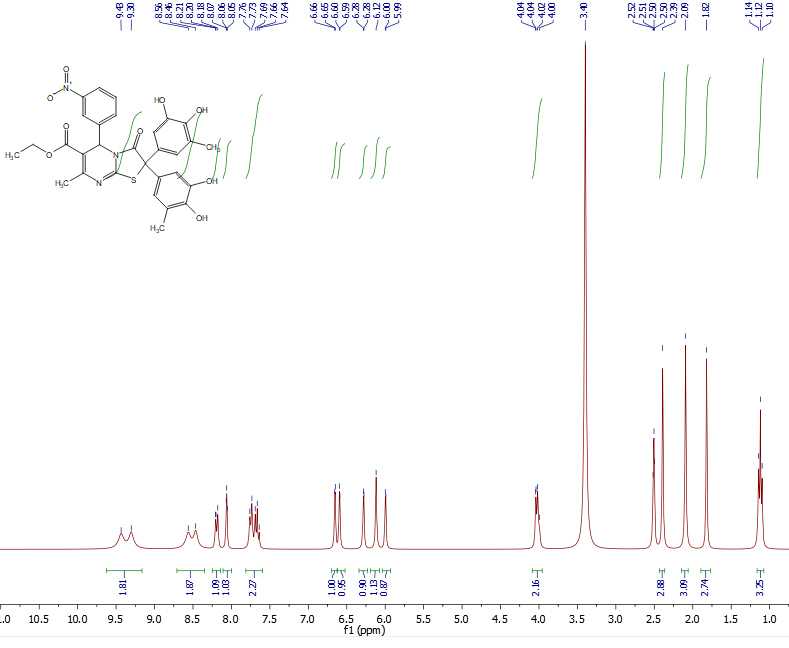


**Figure 52S.** ^1^HNMR (300 MHz, DMSO-*d_6_*) of compound **4o**


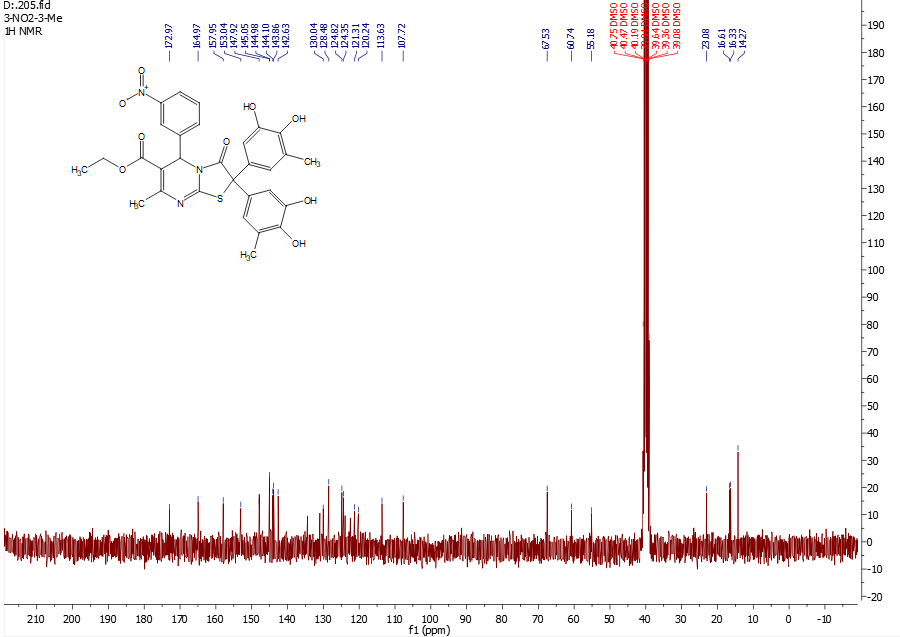


**Figure 53S.** ^13^CNMR (75 MHz, DMSO-*d_6_*) of compound **4o**

605.6


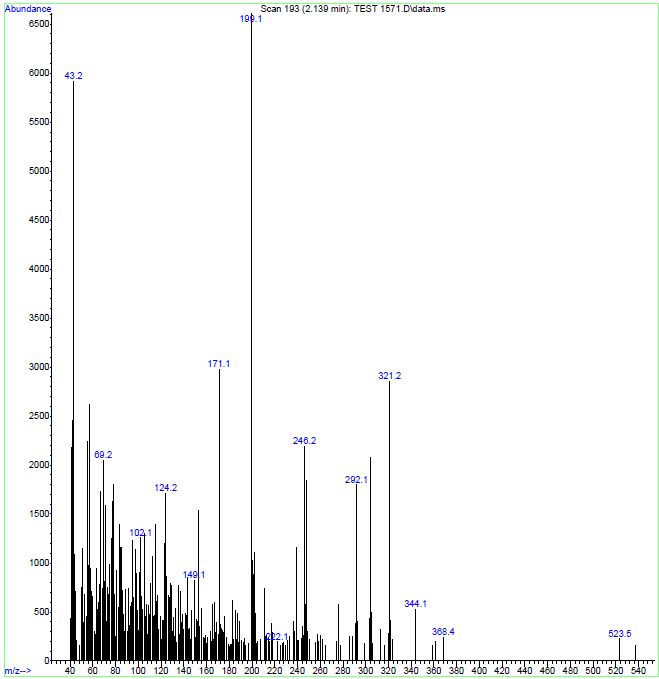


**Figure 54S.** Mass spectra of **4o**
